# Immunological profiling of patients with ulcerative colitis leads to identification of two inflammatory conditions and CD1a as a disease marker

**DOI:** 10.1186/s12967-016-1048-9

**Published:** 2016-11-03

**Authors:** M. Föhlinger, P. Palamides, U. Mansmann, F. Beigel, M. Siebeck, R. Gropp

**Affiliations:** 1 Department of General Visceral, and Transplantation Surgery, Hospital of the LMU Munich, Nussbaumstr. 20, 80336 Munich, Germany; 2 Institute of Molecular Animal Breeding and Biotechnology, and Laboratory for Functional Genome Analysis (LAFUGA), Gene Center, LMU Munich, 81377 Munich, Germany; 3 Institute for Medical Informatics, Biometry and Epidemiology, LMU Munich, Marchioninistr. 15, 81377 Munich, Germany; 4 Department of Medicine II-Grosshadern, Ludwig-Maximilians-University (LMU), Marchioninistr. 15, 81377 Munich, Germany

**Keywords:** Ulcerative colitis, CD1a, HGF, TGFß1, TARC, Immune-profiling, Correlation analysis, Biomarker

## Abstract

**Background:**

Conventional approaches to understand mechanisms underlying the development of pathological manifestations in ulcerative colitis (UC) mostly rely on identification of certain cell types and cytokines followed by verification of their roles in vitro and in vivo. In light of the highly dynamic processes in UC, requiring the cross talk of immune cells, epithelial-, endothelial-, muscle cells and fibrocytes, this approach might neglect temporal and spatial connectivity of individually differing inflammatory responses.

**Methods:**

We undertook a more holistic approach whereby we designed a flow cytometric analysis- and ELISA panel and determined the immunological profiles of UC patients in comparison to Non UC donors. This panel consisted of B-cells, T-cells, macrophages, monocytes, NK- and NK T-cells and subtypes thereof, the cytokines TGFß1 and HGF, the chemokine TARC and periostin. Blood was collected from 41 UC patients and 30 non-UC donors. Isolated PBMC were subjected to flow cytometric analysis and sera were analyzed by ELISA. Data were analysed by cluster- and correlation analysis. To corroborate that the identified cells reflected the inflammatory condition in the colon of UC patients, leucocytes were isolated from colons of UC patients and subjected to the same flow cytometric analysis.

**Results:**

Immunological profiling followed by cluster- and correlation analysis led to the identification of two inflammatory conditions: An ‘acute’ condition characterized by adaptive immune cells as plasma cells,  TSLPR expressing CD11b+ macrophages, CD64 and CCR2 expressing CD14+ monocytes, HGF and TARC and a ‘remodeling’ condition signified by NK T-cells and TLSPR expressing CD14+ monocytes, TGFß1 and periostin. ROC analysis identified TARC and TGFß1 as biological markers with high potential to discriminate between these two conditions (Δ = −6687.72 ng/ml; p = 1E−04; AUC = 0.87). In addition, CD1a+ CD11b+ macrophages (Δ = 17.73% CD1a+ CD11b+; p = 5E−04; AUC = 0.86) and CD1a+ CD14+ monocytes (Δ = 20.35; p = 0.02, AUC = 0.75) were identified as markers with high potential to discriminate between UC and Non UC donors. CD1a+ CD11b+ macrophages and NK T-cells were found to be significantly increased in inflamed colons of UC patients as compared to non-UC control samples (p = 0.02).

**Conclusions:**

Immunological profiling of UC patients might improve our understanding of the pathology underlying individual manifestations and phases of the disease. This might lead to the development of novel diagnostics and therapeutic interventions adapted to individual needs and different phases of the disease. In addition, it might result in stratification of patients for clinical trials.

**Electronic supplementary material:**

The online version of this article (doi:10.1186/s12967-016-1048-9) contains supplementary material, which is available to authorized users.

## Background

Ulcerative colitis (UC) belongs to the chronic inflammatory diseases of unknown etiology. Manifestations of the disease differ highly with regard to onset, severity, course and response to therapeutics and it is likely, that various forms of the disease are covered by the umbrella diagnosis of UC. It is presently thought that a combination of genetic, environmental and microbiotic factors is responsible for an uncontrolled immune response characterized and driven by the typical Th2 cytokine Interleukine (IL)-13 (for review see [1]). We have recently taken a slightly different view which is based on the assumption that the inflammatory response partially resembles an uncontrolled wound healing process caused by epithelial damage [2]. In this model an active role is assigned to epithelial cells as they release damage associated molecular pattern molecules (DAMP) such as TSLP (thymic stromal lymphopoietin) to direct immune cells towards a Th2 characterized immune response ultimately resulting in increased mucus production, epithelial cell hyperplasia and fibrosis. DAMPs comprise heterogeneous molecular entities ranging from toxic agents such as uric acid to proteases and allergens. The release of TSLP by epithelial cells can be induced by damage, sensing microbial products or dsRNA via toll like receptors (TLR) [3–9]. This results in macrophages, dendritic cells (DC) and monocytes having the center stage because as the preferred targets of TSLP and as a highly volatile cellular population they have the capacity to further fuel or dampen inflammation depending on the inflammatory milieu. The crucial role of macrophages in UC is supported by the efficacy of infliximab an anti-tumor necrosis factor (TNFα) monoclonal human antibody with IgG1 effector function which is thought to be deleterious to macrophages expressing TNFα on their surfaces [10].

Wound healing processes are also governed by macrophages. Here, neutrophils and inflammatory macrophages (M1) infiltrate damaged tissue as a first step to protect the organism from pathogens. Once the wound is sealed macrophages engulf apoptotic neutrophils in a process called efferocytosis and in consequence alter their phenotype from inflammatory to healing (M2) [11, 12]. Macrophages are main sources of hepatic growth factor (HGF), transforming growth factor (TGF) ß1 and thymus and activation regulated chemokine (TARC), all of which play a crucial role in wound healing processes and have been described as elevated in biopsies and/or sera of UC patients [13, 14]. Hepatic growth factor induces re-epithelization and is expressed in fibroblast, monocytes, dendritic- and endothelial cells. HGF leads to proliferation, motility, IL-1ß, IL-4, GM CSF secretion and suppresses CD4+ and CD8+ cells [15]. TGFß1 induces fibrosis and scarring and additionally acts as an immune modulator. It is expressed in regulatory T-cells, M2 macrophages, fibroblasts and platelets [16, 17]. TARC is expressed in epithelial cells, monocytes and macrophages and attracts CCR4 bearing Th2 cells, regulatory T-cells and macrophages into the damaged tissue [18, 19]. Finally, periostin is a protein associated with remodeling of the colon and lung architecture and expressed under the control of IL-13 which has been identified as a biomarker for patients responding to IL-13 therapy [20, 21]. Expressed by epithelial cells and fibroblasts and acting in an autocrine manner it induces collagen disposition, TGFß1 secretion, cell migration and epithelial to mesenchymal transition.

CD1a has been known for decades as a phenotypic marker of human epidermal Langerhans cells (LC). Like other members of the CD1 family CD1a presents lipids to evoke T cell activation resulting in the release of IL-22, IL-13 and interferon (IFN)γ [22, 23]. In the healthy skin auto reactivity is thought to be prevented by physical segregation of LC located in the epidermis and the corresponding lipid ligands concentrated in the stratum corneum. Upon injury or inflammation segregation breaks down and LC activate autoreactive T-cells, notably Th22 cells which are the major source of IL-22 production, which is thought to be instrumental in wound healing processes and antimicrobial defense [24–26]. The supply of fatty acid ligands can also be provided by phospholipase A2 (PLA2) an important component of inflammation and bee- and wasp venom [27]. PLA2 releases fatty acids from phospholipids in the extracellular space where loading of CD1a takes place. To date, CD1a bearing macrophages have not been detected in the colon.

To test our hypothesis of UC resembling an uncontrolled wound healing process we analyzed the immunological profile of UC patients and compared these to those of Non UC donors by determining the frequency of immune cells in the blood and serum levels of TARC, HGF, TGFß1 and periostin. Cluster- and correlation analysis was performed to portray individual profiles and to understand the correlation of factors and immune cells in the inflammatory milieu of UC patients. In a second step, we analyzed immune cells isolated from colons of UC patients undergoing colectomy to verify that the cells identified as relevant in the blood reflected the inflammatory condition in the colon.

## Methods

### Ethical considerations

All donors gave informed written consent and the study was approved by the Institutional Review Board (IRB) of the Medical Faculty at the University of Munich (2015–22).

Colon samples and annotated data were obtained and experimental procedures were performed within the framework of the non profit foundation HTCR, including the informed patient’s consent [28].

### Isolation of PBMC

Peripheral blood was collected from the arm vein of patients suffering from UC and Non UC donors. The Non UC donors had no apparent infection, inflammation and did not suffer from other chronic inflammatory diseases. As we could not exclude hidden infections they were considered as Non UC. 10 ml of blood in trisodium citrate solution were diluted with Hank’s balanced salt solution (Thermofisher, Waltham, USA) in a 1:2 ratio and 30 ml of the solution loaded onto Leukosep tubes (Greiner Bio One, Frickenhausen, Germany). Cells were separated by centrifugation with 800*g* for 30 min. The interphase containing PBMC was in Hanks balanced salt solution and centrifuged with 1400*g* for 5 min. The cell pellet was resuspended in sterile phosphate-buffered saline (PBS) at a concentration of 4 × 10^6^ cells in 100 µl.

### Isolation of lamina propria leucocytes

For isolation of lamina propria mononuclear cells (LPMC) from human colons which were extracted from UC and colon cancer patients, a protocol modified of Hansson et al. [29] was used. A piece in the size of approximately 2 × 2 cm of colonic wall were kept in 1× RPMI (Thermofisher, Waltham, USA) containing 10% FCS on ice until preparation. The mucosa was dissected of underlying muscular layers and fat with scissors and cut into small pieces <5 mm.

The tissue was predigested for 4 × 15 min in 15 ml predigestion solution containing 1× HBSS (Thermofisher, Waltham, USA), 5 mM EDTA, 5% FCS, 100 U/ml Pencillin–Streptomycin (Sigma-Aldrich Co.,St. Louise USA) in an orbital shaker with slow rotation (40 g) at 37 °C. To remove epithelial cells, cell suspensions were filtered through a nylon filter. Following removal of excess EDTA with RPMI the pieces were cut into finer pieces of <1 mm and digested for 60 min in digestion solution containing 1× RPMI, 10% FCS, 1 mg/ml collagenase A (Sigma-Aldrich Co., St. Louise USA), 10 KU/ml Dnase I (Sigma-Aldrich Co., St. Louise USA), 100 U/ml Pencillin–Streptomycin (Sigma-Aldrich Co., St. Louise USA) in an orbital shaker with slow rotation (40 g) at 37 °C.

Isolated LPMC were collected by centrifugation with 177* g* for 10 min and resuspended for FACS analysis. Cell suspensions were filtrated one more time using a 35 µm cell strainer for further purification before labelling the cells for flow cytometry analysis.

### Flow cytometric analysis

Cellular markers to phenotype UC patients and healthy controls are depicted in Table 1.

**Table 1:** Cellular markers used in phenotyping of UC patients

Marker	Definition	References
CD19+ CD27+ IgD+	Unswitched memory B-cell	[30]
CD19+ CD27+ IgD−	Switched memory B-cell	[30]
CD19+ CD38+	Plasma cell	[30]
CD4+ CD25+	Activated CD4+ T-, regulatory T cells, Th2 cells	[31, 32]
CD14+	MC	[33]
CD14+ CD80+ CD86+	MC, mature	[33–35]
CD14+ CCR2+	MC, tissue penetrating, inflammatory	[36]
CD14+ TSLPR+	MC, expressing TSLPR	
CD14+ CD64+	M1MC	[37]
CD14+ CD163+	M2 MC, scavenging cells	[35]
CD14+ CD1a	MC CD1a expressing	
CD11b+	cDC1	[33]
CD11b+ CD80/86+	cDC1, mature	
CD11b+ CD1a+	cDC1 CD1a expressing	
CD11b+ TSLPR+	cDC1 TSLPR expressing	
CD3+ CD56+	NK T-cell	[38]
CD3− CD56+	NK cell	
CD3− CD56−, CRTH2+ CD127+	ILC2	[39–41]

*MC* monocyte, *NK* natural killer cell, *TSLPR* thymic stromal lymphopoietin protein, *cDC* conventional dendritic cell

**Table 3:** Immunological profiling leads to identification of specific cellular markers for UC and to markers signifying therapeutic responses

Leukocytes [% FoP]	UC/Non UC			TNFa-blocker/all other			Glucocorticoid/all other			Mesalazine/all other			Immunosuppressiva/all other		
	Δ	p value	95% CI	Δ	p value	95% CI	Δ	p value	95% CI	Δ	p value	95% CI	Δ	p value	95% CI
CD19+	−*8.80*	*0.05*	−*0.1* to *17.7*	−10.78			*24.07*	*0.01*	*5.1* to *43.0*	−3.99			*25.85*	*0.05*	−*1.0* to *52.78*
CD19+ CD27+ IgD+	−6.07			5.50			−*13.67*	*0.009*	−*23.74* to −*3.58*	6.47			−*16.27*	*0.006*	−*27.45* to −*5.08*
CD19+ CD27+ IgD−	10.57			3.47			*18.65*	*0.002*	*7.0* to *30.27*	−1.56			*20.36*	*0.002*	*7.82* to *32.88*
CD19+ CD38+	−*37.10*	*5E−03*	*23.4* to *52.5*	−*29.82*	*8E*−*04*	−*45.8* to *−13.2*	14.75			−4.10			*27.99*	*0.05*	*0.46* to *55.5*
CD4+	−*8.56*	*0.006*	*2.7* to *14.9*	2.00			−2.71			−6.64			*8.63*	*0.03*	*0.8* to *16.4*
CD4+ CD25+	−*7.16*	*3E−05*	*4.1* to *10.4*	−2.20			−0.68			−2.64			5.53		
CD8+	−0.19			−4.75			1.30			0.97			*11.60*	*0.008*	*4.0* to *19.78*
CD11b+	−*6.31*	*0.005*	*2.1* to *11.2*	−4.12			2.85			−2.93			−0.86		
CD11b+ TSLPR+	3.73			−*15.36*	*0.04*	−*30.26* to −*0.44*	*23.92*	*0.03*	*2.4* to *45.3*	0.08			23.55		
CD11b+ CD1a+	*17.73*	*5E*−*04*	−*27.9* to *−8.3*	4.10			−12.65			8.76			−6.59		
CD11b+ CD80/86+	−0.97			−*12.86*	*0.04*	−*25.3* to *−0.35*	0.38			9.17			3.95		
CD14+	−0.14			−0.86			0.37			−0.83			−0.67		
CD14+ CCR2+	15.58			−5.78			11.75			−1.05			1.59		
CD14+ CD80/86+	−10.69			8.03			−*26.71*	*0.03*	−*49.95* to *−3.4*	−2.07			−20.97		
CD14+ TSLPR+	*3.53*	*0.03*	−*8.4* to *−0.5*	*6.74*	*0.03*	*0.8* to *12.4*	1.67			1.79			3.00		
CD14+ CD1a+	*20.35*	*0.02*	−*40.8* to -*4.5*	6.91			4.38			6.40			3.76		
CD14+ CD64+	−*9.87*	*0.06*		−0.14			−0.51			−0.66			0.50		
CD14+ CD163+	−*3.54*	*0.05*	*6.0* to *2.5*	0.15			−2.03			0.00			−0.99		
CD3− CD56+	−0.65			−0.58			1.80			−2.59			4.39		
CD3+ CD56+	*6.35*	*0.001*	−*10.3* to −*2.6*	−1.41			−4.94			3.22			−4.36		
Factors [µg/ml]															
TARC	1.79			−*6.69*	*1E−04*	*32.57* to *99.45*	4.25			−1.34			5.91	*3E−03*	*23.92* to *94.34*
HGF	*1.41*	*6E−04*	−*21.98* to −*0.62*	*2.02*	*0.001*	−*32.20* to −*0.82*	*2.05*	*0.03*	*0.18* to *39.11*	−0.11			1.63		
TGFß1	*16.76*	*3E−03*	*23.53* to *38.14*	−*22.13*	*0.01*	*52.94* to *30.81*	−*24.15*	*0.03*	−*45.31* to −*29.97*	−4.36			−26.47	*0.05*	−*53.10* to −*0.16*
Periostin	−13.43			−21.17			−*29.87*	*0.02*	−*53.84* to −*59.07*	−1.47			−17.06		

PBMC and serum from UC patients and Non UC patients were isolated and subjected to flow cytometric analysis. The table displays differences of mean values of frequency of immune cells in the blood and serum levels of factors. UC patients versus Non UC donors, UC patients treated with TNFa blocker, Glucocorticoid, Mesalazine and Immunesupprssiva compared to all other patients (complete data set in Additional file 1: Table S1)

A two-sided t-test and a significance level = 0.05 was used to compare groups. Differences in mean values = Δ. *CI* confidence interval. Significantly altered values are depicted in italic numbers

**Table 4:** Leucocytes characterizing the two inflammatory conditions

Leucocytes	Acute inflammatory	Remodeling inflammatory
CD19+ CD27+ IgD+	−	+
CD19+ CD27+ IgD−	+	−
CD19+ CD38+	+	−
CD4+ CD25	+	
CD11b+ TSLPR+	+	−
CD11b+ CD1a+		+
CD14+ TSLPR+	−	+
CD14+ CCR2+	+	−
CD14+ CD1a+	?	?
CD14+ CD64+	+	
CD3+ CD56+		+
CD3− CD56− CD294+ CD127+		+
Factors [ng/ml]		
TARC	+	−
HGF	+	−
TGFß1	−	+
Periostin	−	+

Labelling of human leukocytes was performed as described in Table 1. All anti human antibodies were purchased from Biolegend (San Diego, USA) and used according to manufacturer’s instructions. Samples were measured using a BD FACS Canto II™ and analysed with FlowJo 10.1-Software (FlowJo LLC, Oregon, USA).

### ELISA analysis

Human serum TARC, HGF, TGFß1, periostin, levels were measured via Enzyme linked Immunosorbent Assay (ELISA) (Biotechne, Minneapolis, USA) according to the manufacturer’s instructions. Sample was measured in duplicates.

### Statistical analysis

Statistical analysis was performed with R: A language and environment for statistical computing. R Foundation for Statistical Computing, Vienna, Austria (3.2.2). URL https://www.R-project.org/. Variables were represented with mean, standard deviation, median, and IQR values. A two-sided t.test and a significance level = 0.05 was used to compare two groups and for more than two groups ANOVA followed by TukeyHSD was conducted. Correlation analysis was performed using spearman rank correlation analysis. ROC curves were performed using R. Heatmap was performed using R (default).

## Results

### Immuno-profiling of UC patients

In order to characterize the inflammatory condition, blood samples were collected from UC patients and Non-UC patients (Table 2). Peripheral blood mononuclear cells (PBMC) and sera were collected as described in “Methods” section and samples were subjected to flow cytometric analysis to determine the frequencies using surface markers of B-cells, T-cells, macrophages, monocytes, dendritic cells, NK T- and NK cells and subtypes thereof (Table 1; gating strategy Additional file 1: Fig. S1).

**Table 2:** Baseline demographics, duration of disease and therapy

	UC N = 41	Non UC N = 30
Age (years)		
Mean (SD)	39.3 (18.8)	28.9 (11.0)
Range	24–60	21–62
Gender, male, n (%)	21 (50)	7 (25)
Duration of UC (years)		
Mean (SD)	11.42 (9.43)	
Range	1–37	
Treatment (current)		
TNFα-blocker	21	
Glucocorticoids	11	
Mesalazine	24	
Immuno-suppressive	7	
No	4	

As TARC, HGF, TGFß1 and periostin are considered important factors of wound healing processes serum levels of these factors were also quantified.

As shown in Table 3 (complete data set in Additional file 1: Table S1) a significant decline in frequencies was found plasma-cells, CD4+ and activated CD4+ CD25+ cells, CD11b+ macrophages, CD11c+ dendritic cells in the patient group as compared to the Non UC group. In contrast, increased frequencies of CD1a expressing CD11b+ macrophages, TSLPR-, CD1a-, and CD163 expressing CD14+ monocytes and NK T cells were observed. In addition, serum levels of HGF and TGFß1 were increased in the UC group.

As only a few therapeutically naïve patients participated in the study and as TNFα-blockers, glucocorticoid, mesalazine and immune-suppressors are supposed to suppress or modulate immunological responses albeit in different ways, these results did not necessarily suggest that the observed differences were disease specific. Therefore, patients were further subdivided into groups treated with TNFα-blockers, glucocorticoids, mesalazine and immune-suppressing drugs and compared to the rest of the patient group. As shown in Table 3 treatment with the respective therapeutics affected frequencies of immune cells and serum concentrations of factors and resulted in opposing affects. In contrast to patients treated with glucocorticoids, mesalazine and immune-suppressors, patients treated with TNFα-blockers displayed lower frequencies of cells of the adaptive immune system to include plasma-cells, TSLPR+ and mature CD11b+ macrophages and dendritic cells along with a decline in HGF and TARC, whereas frequencies of TSLPR+ CD14+ monocytes and serum levels of TGFß1 increased. Opposing effects were observed in the patient group treated with glucocorticoids. In this group, cells of the adaptive immune system were not suppressed. Frequencies of B-cells, switched memory B-cells, plasma cells and TSLPR expressing CD11b+ macrophages displayed increased frequencies along with and HGF and TARC serum levels. Unswitched CD19+ CD27+ IgD+ B-cells, mature CD14+ monocytes, TGFß1- and periostin serum levels decreased in this group. Of note, patients treated with TNFα blockers did not regain the profile of Non UC patients.

In the mesalazine treated group a decline of CD11c dendritic cells was observed. Like in the group treated with glucocorticoids, the group treated with immune-suppressive drugs no negative affect was observed on B-cells and subtypes thereof. However, a decline in CD8+ T-cells, mature CD14+ monocytes was detected along with decreased TGFß1 serum levels. In contrast TARC serum levels were increased.

**Fig. 1:**
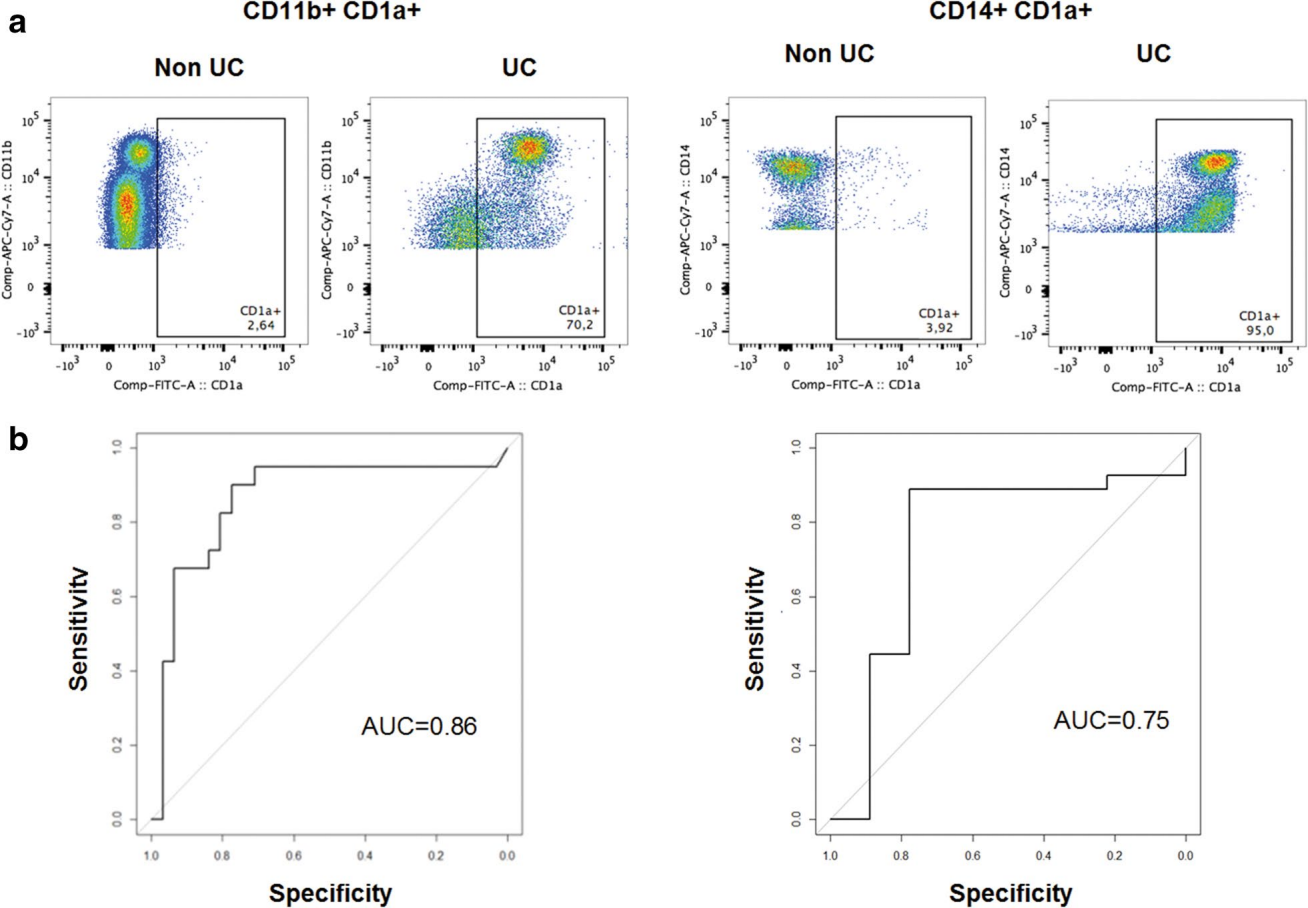
CD1a expressing macrophages and monocytes as disease specific markers. **a** Exemplary flow cytometric analysis of CD11b+ CD1a and CD14+ CD1a populations in PBMC from a Non UC donor and a UC patient. **b** ROC curve for CD11b CD1a+ (Non UC n = 31, UC n = 40) and CD14+ CD1a (Non UC n = 9, UC n = 27) in differentiating UC and Non UC patients

As CD11b+ CD1a+ and CD14+ CD1a+ seemed to be a cell population discriminating between Non UC and UC and frequencies of these cell types were unaffected by therapeutic interventions a ROC analysis was performed to evaluate the quality of this marker (Fig. 1). The analysis revealed a high specificity and sensitivity for CD1a expressing CD11b+ and CD14+ macrophages.

As in the UC group the disparity in age and duration of the disease was high, a correlation analysis was performed to exclude that age and/or duration of disease were responsible for observed differences. And indeed, antigen unexperienced CD8+ T-cells defined as CD44- cells correlated negatively with age whereas antigen experienced T-cells (CD44+), CD11b and NK T-cells correlated positively with age (Additional file 1: Fig. S2). This observation is in agreement with previous data which also showed a decline of naïve T-cells and accumulation of antigen-experienced T-cells [40]. Thus, the age effect might affect observations obtained from analyzing T-cells. With the exception of NK T-cells all of these cell types also correlated with duration of disease, indicating both parameters might affect these cell types.

**Fig. 2:**
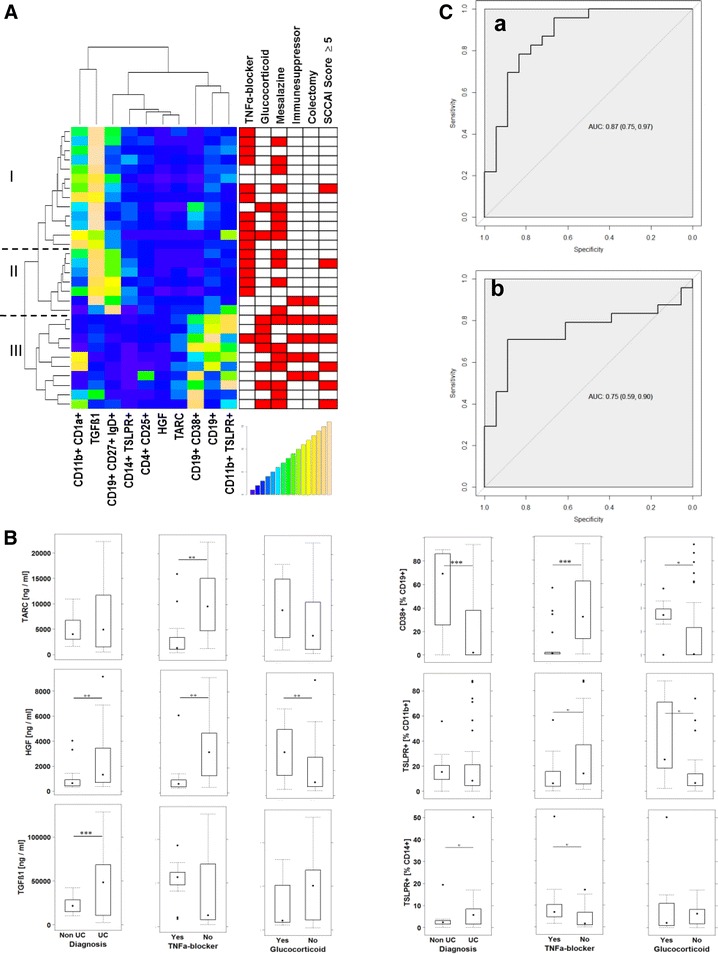
Immunological profiling leads to identification of distinct subgroups of UC patients. PBMC and serum were isolated from UC patients and subjected to flow cytometric analysis and ELISA, respectively. **A** Hierachically clustered heatmap based on frequencies of immune cells and serum levels of TGFß1, HGF and TARC. (linkage hierarchical cluster with euclidean distance). Simple clinical colitis activating index (SCCAI). **B** Boxplot analysis of concentrations of TARC, HGF and TGFß1 and plasma cells and TSLPR expressing CD11b+ macrophages and monocytes in dependence of diagnosis or treatment with TNFα-blockers and glucocorticoids. (UC patients n = 30, treated with TNFα blocker n = 16, Glucocorticoid n = 9, mesalazine n = 16, colectomized patients n = 5. **C** ROC curve for TARC (*a*) and TGFß1 (*b*) in differentiating group I+ II and III. TARC: 95% CI value 0.74–0.97, 18 cases treated with TNFα-blocker, 23 cases no treatment with TNFα-blockers; TGFß1: 95% CI value: 0.59–0.9, 18 cases treated with TNFα-blocker, 24 cases no treatment with TNFα-blockers

In order to examine whether the selected immune-cells and markers could serve to identify individual signatures resulting in defining subgroups of UC patients, collected data were further subjected to a cluster analysis and the result was displayed as a heatmap. As shown in Fig. 2a UC patients clustered in three main groups. Group I was signified by high levels of CD11b+ CD1a+, TGFß1, unswitched B-cells, TSLPR expressing CD14+ monocytes. In contrast, HGF and TARC, plasma cells, CD19+ B-cells, TSLPR expressing CD11b+ macrophages were decreased. Group III displayed a counter image to this pattern and group II represented an intermediate pattern between group I and III. Patients treated with TNFα-blockers mainly clustered in group I and II, whereas patients treated with glucocorticoids were mainly detected in group III. Patients treated with mesalazine were evenly spread in all three groups and patients treated with immune-suppressing drugs were found in group II and III.

Patients with a simple clinical colitis activating index (SCCAI) ≥5 [41, 42] were considered as patients experiencing a relapse. The fact that these patients mainly clustered in group III suggested less efficacy of glucocorticoids, mesalazine and immune-suppressing drugs as compared to TNFα-blockers and that the profile associated with patients in group III reflected a more active form of the disease than response to therapeutics. Boxplot analysis of the main discriminators corroborated the findings of the heatmap (Fig. 2b). TARC, HGF and TGFß1 expression as well as frequencies of plasma cells and TSLPR expressing CD11b+ macrophages and CD14+ monocytes were affected by treatment with TNF-α-blockers.

The expression and cluster analysis suggested that TARC and to a lesser degree TGFß1 could serve as a marker to discriminate between the active form and the form in remission. ROC analysis corroborated this observation and identified TARC and TGFß1 as markers with high potential to discriminate between the two inflammatory conditions (Fig. 2c).

**Fig. 3:**
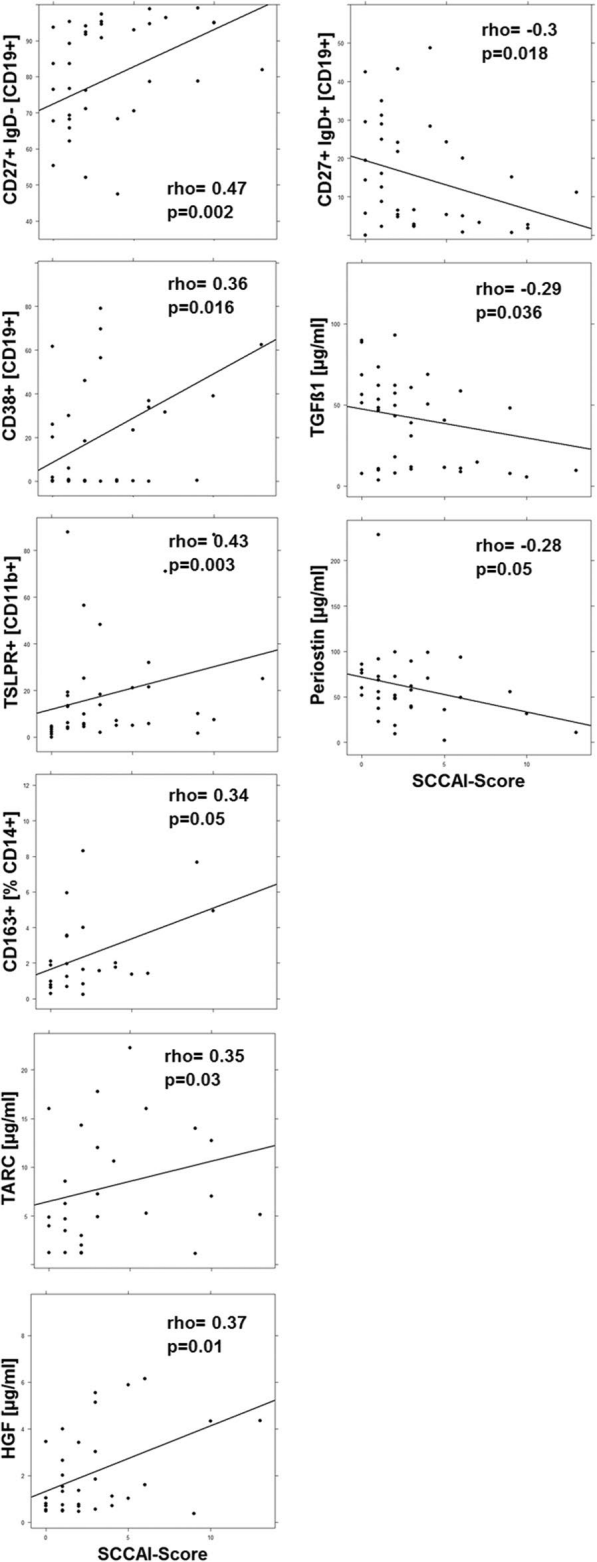
Correlation analysis of the clinical activity score (SCCAI) with subtypes of immune cells and serum factors depicted as scatter plots. (method = Spearman, numbers display Spearman rank-order correlation coefficients (rho-values) and p values

**Fig. 4:**
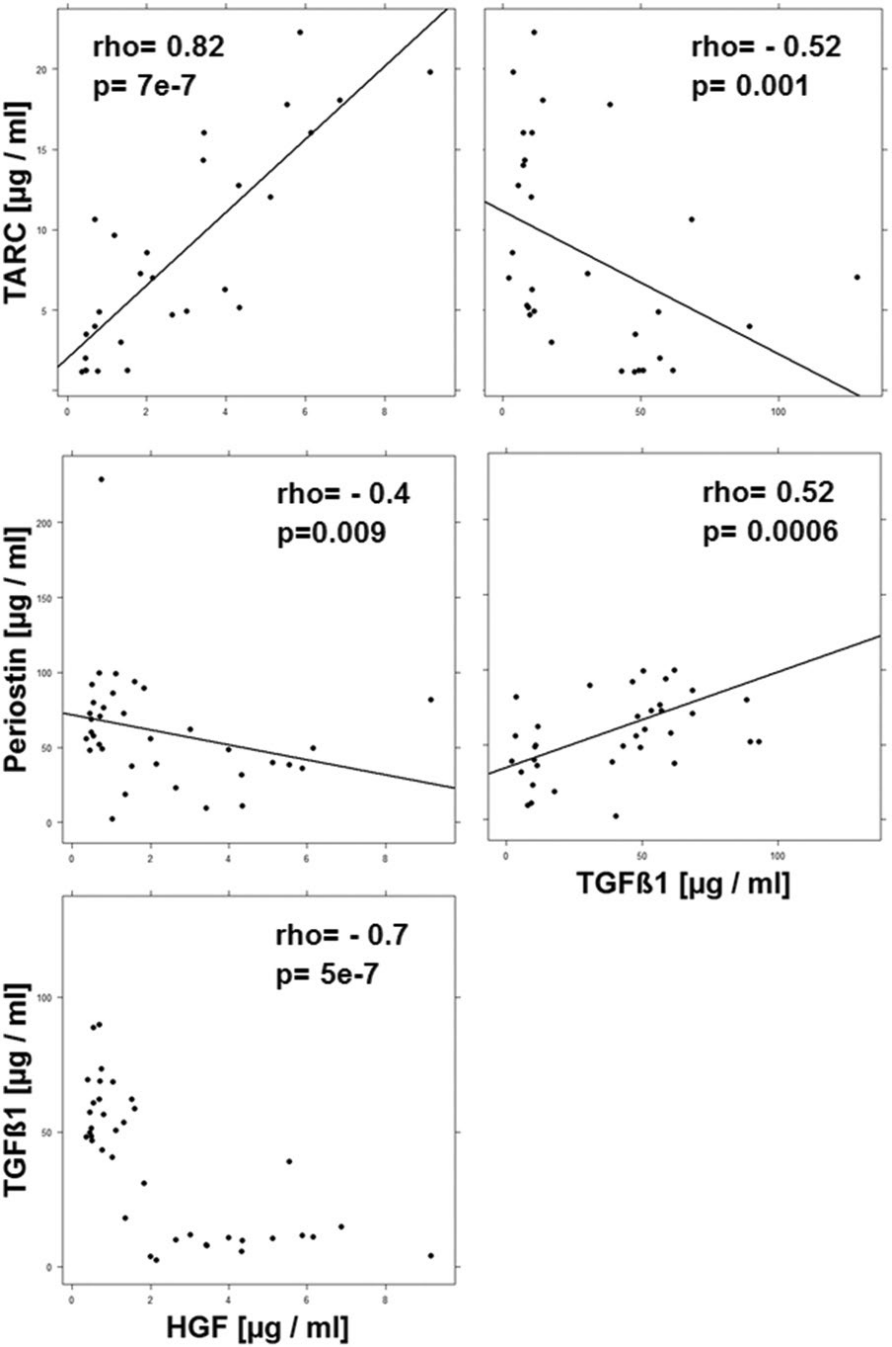
Correlation- and interrelation analysis of TARC, HGF, TGFß1 and periostin depicted as scatter plots. (method = Spearman, numbers display Spearman rank-order correlation coefficients (rho-values) and p values

**Fig. 5:**
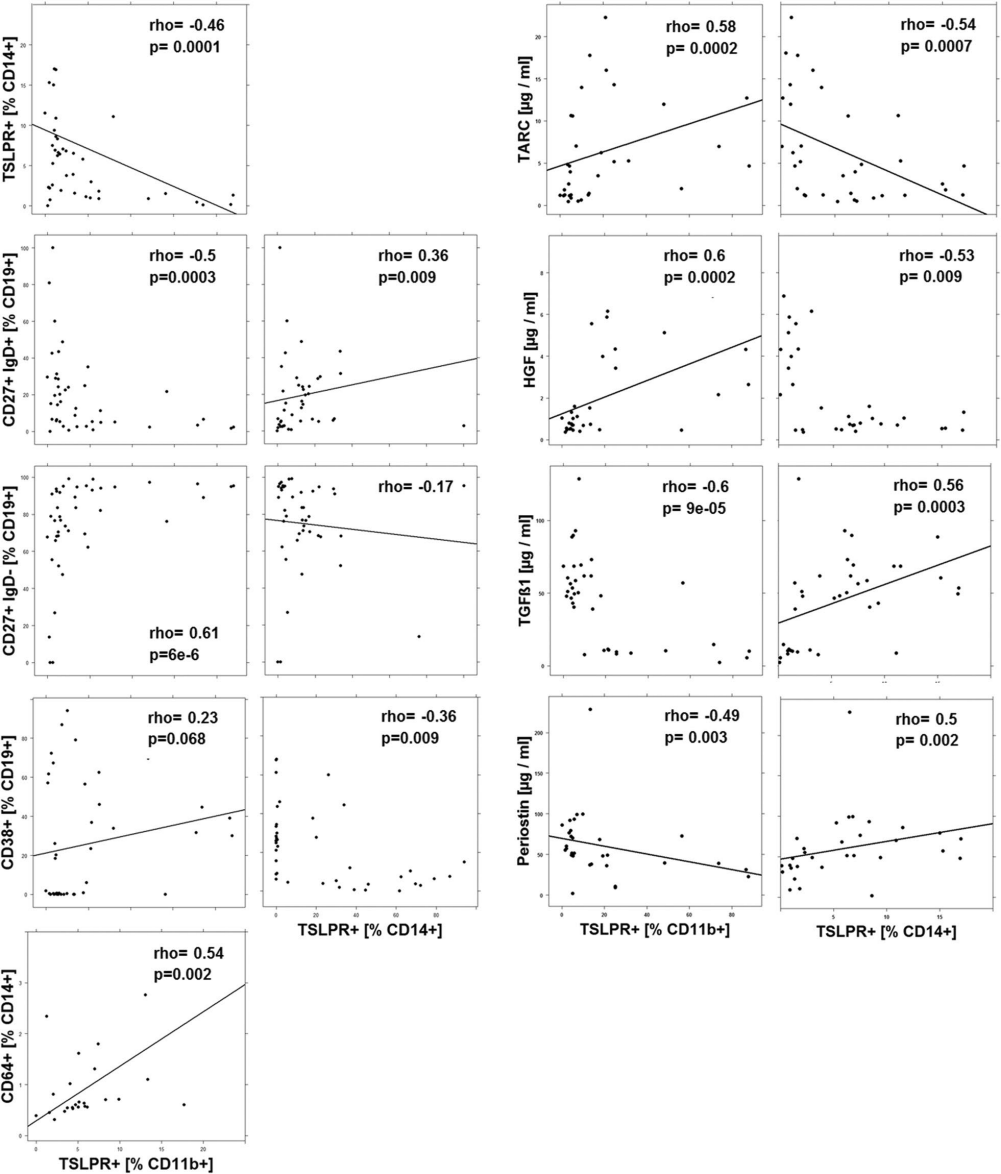
Correlation- and interrelation analysis of CD11b+ TSLPR+ macrophages and CD14+ TSLPR+ monocytes with subtypes of immune cells and serum factors depicted as scatter plots. (method = Spearman, numbers display Spearman rank-order correlation coefficients (rho-values) and p values

**Fig. 6:**
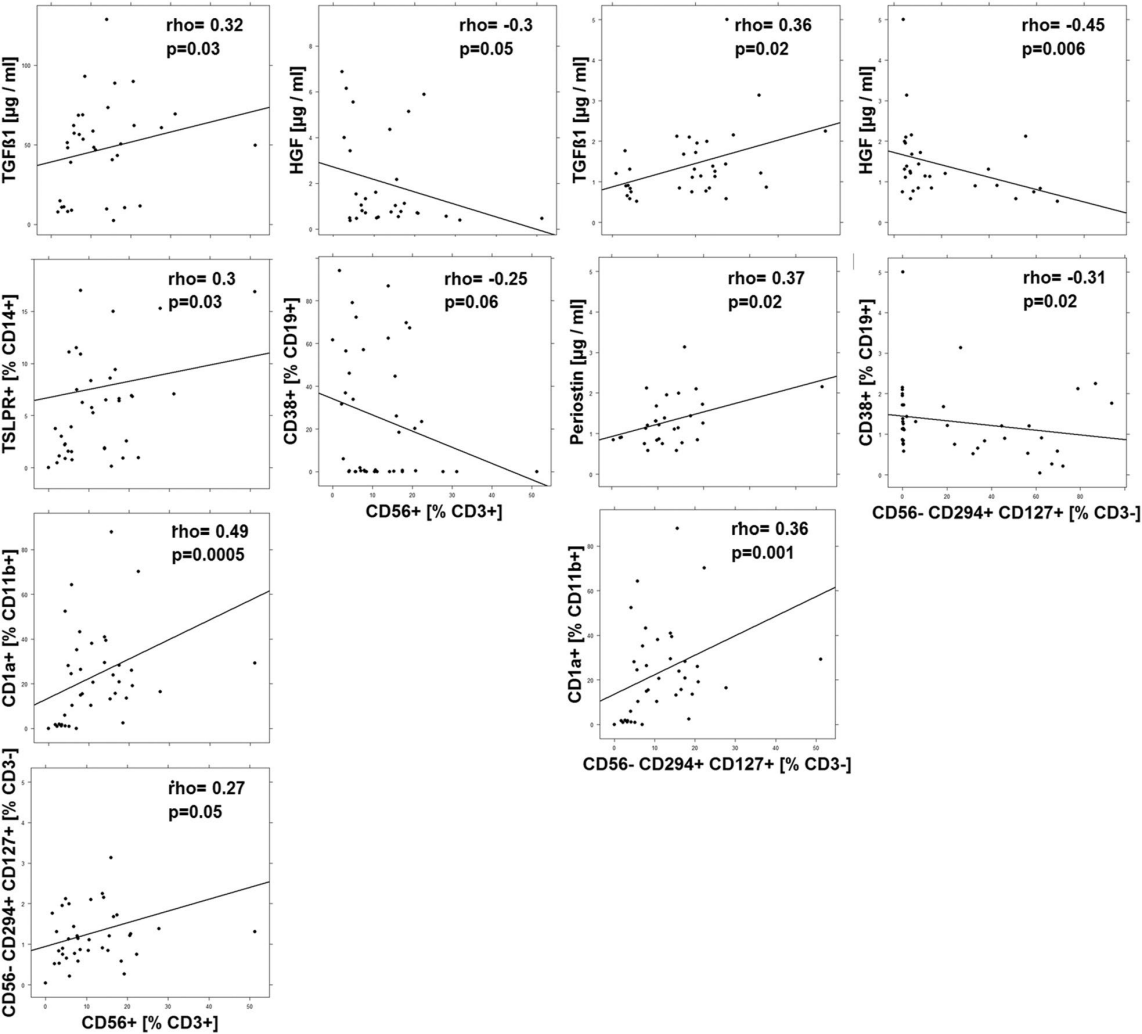
Correlation- and interrelation analysis of NK T-cells and ILC2 with subtypes of immune cells and serum factors depicted as scatter plots. (method = Spearman, numbers display Spearman rank-order correlation coefficients (rho-values) and p values

**Fig. 7:**
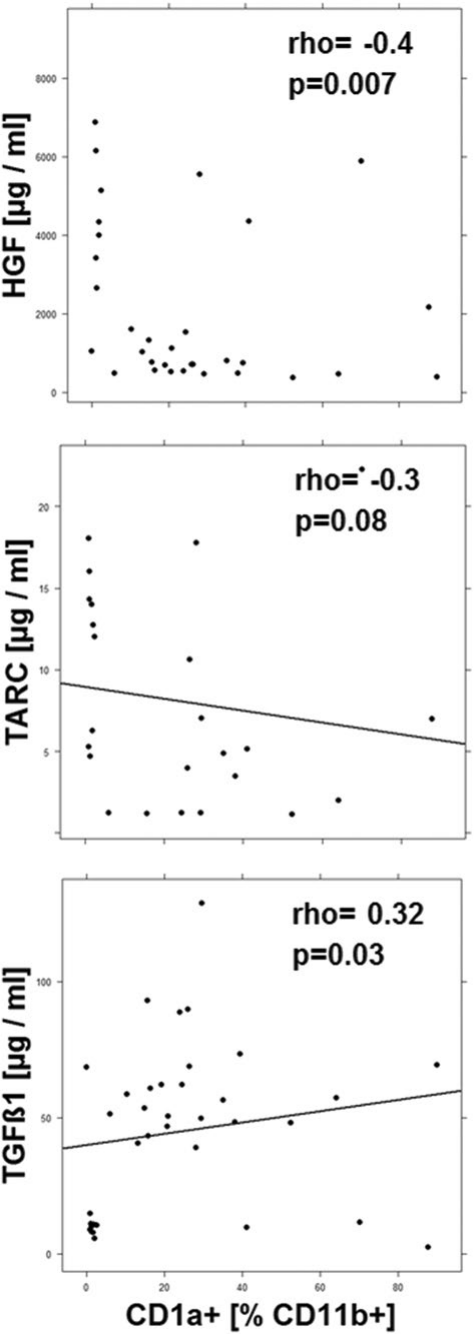
Correlation analysis CD11b+ CD1a+ macrophages with serum factors depicted as scatter plots. (method = Spearman, numbers display Spearman rank-order correlation coefficients (rho-values) and p values

As the profiling and cluster analysis suggested that mainly two immunological profiles prevailed in the UC patient group a correlation analysis was performed (Figs. 3, 4, 5, 6, 7). In addition to the examined immune-cells, the cluster analysis included the clinical activity score (SCCAI-Score) [41] and HGF, TARC, TGFß1 and periostin.

#### Correlation analysis of the SCCAI-Score with immune cells and serum factors

As shown in Fig. 3 the SCCAI Score positively correlated with switched memory B-cells, plasma cells, TSLPR+ macrophages, M2 resident monocytes, HGF and TARC, whereas it negatively correlated with unswitched memory B-cells, TGFß1 and periostin, suggesting that the adaptive immune cells along with TSLPR expressing macrophages play an important role during the active form of the disease and that HGF and TARC are hallmarks of this condition. In contrast, TGFß1 and periostin signified a second inflammatory condition which—due to the molecular function of the proteins—most probably was characterized by remodeling or the colon architecture.

#### Correlation- and interrelation analysis of serum factors

To corroborate these observations correlation and interrelation analysis was performed with these serum markers. As shown in Fig. 4 HGF and TARC were positively correlated with each other and both factors were negatively correlated with TGFß1 and periostin. Likewise, TGFß1 and periostin were positively correlated with each other further indicating that elevated or decreased concentrations of HGF/TARC and TGFß1/periostin reflected different inflammatory conditions.

#### Correlation- and interrelation analysis of TSLPR expressing macrophages and monocytes with immune cells and serum factors

TSLP is thought to be an important cytokine to promote a Th2 characterized inflammation in response to epithelial damage. Monocytes and dendritic cells exhibit the highest expression of the TSLP receptor (TSLPR) and are therefore the preferred targets of TSLP. Measurement of TSLP in serum of UC patients in comparison to healthy subjects revealed a decline in TSLP concentrations in UC patients (data not shown), suggesting that either TSLP does not play a role in UC or monocytes and macrophages cells act as ‘TSLP sinks’. The strong therapeutic response to TNFa-blockers in CD11b+ TSLPR+ macrophages (Table 3), however, supported the latter idea.

TSLPR is expressed on CD11b+ macrophages and CD14+ monocytes, and as shown in Fig. 5 both cell types correlated negatively with each other. This pattern was preserved with regard to unswitched B-cells, switched B-cells, plasma cells, M1 monocytes and the expression of TARC, HGF, TGFß1 and periostin. Whenever TSLPR+ expressing CD11b+ macrophages correlated positively, TSLPR+ expressing CD14+ monocytes correlated negatively and vice versa.

#### Correlation- and interrelation analysis of NK T-cells and ILC2 with immune cells and serum factors

NK T-cells are considered a major source of IL-13 which is thought play a key role in inducing pathological manifestations in UC. In order to examine whether NK T-cells can be assigned to the identified inflammatory conditions these cells were also subjected to correlation analyses. As shown in Fig. 6 NK T-cells correlated positively with CD14+ TSLPR+ monocytes and TGFß1 which were both considered as markers of the ‘remodeling condition’. Conversely, NK T-cells correlated negatively with HGF and plasma cells which are considered as markers for the ‘acute’ condition. The positive correlation of NK T-cells with CD1a expressing CD11b+ macrophages might indicate that these cells also play a role in the remodeling of the colon architecture. ILC2 cells are thought as further source of IL-13 and IL-4 and as these cells also correlated positively with NK T-cells we hypothesized that ILC might act similarly as NK T-cells. And indeed, the correlations reflected the correlations of NK T-cells. Like NK T-cells ILC2 correlated positively with TGFß1, CD1a+ CD11b+ macropohages and additionally with periostin, and like NK T-cells ILC2 correlated negatively with HGF and plasma cells.

#### Correlation analysis of CD1a expressing macrophages serum factors

The correlations of CD11b+ CD1a macrophages with NK T-cells and ILC2 suggested an association of this cell type with the remodeling conditions. To corroborate this assumption, correlations of CD1a expressing CD11b+ macrophages were further analyzed. As shown in Fig. 7 this cell type correlated negatively with HGF and TARC and positively with TGFß1, indicating that they might be involved in the remodeling condition. No correlations could be detected with CD14+ CD1a expressing monocytes.

### The inflammatory condition in the colon of UC patients

**Fig. 8:**
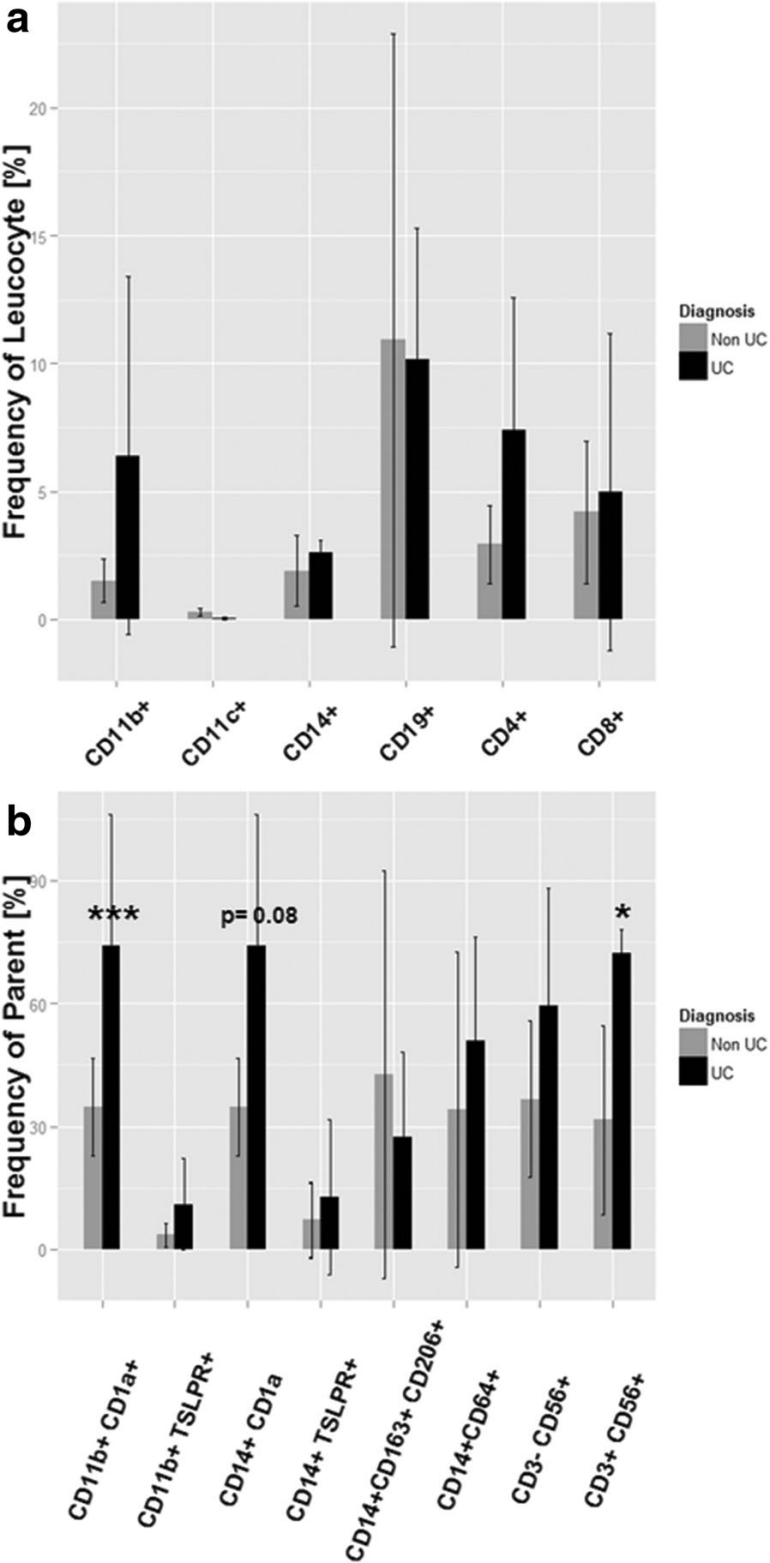
Comparison of frequency of leucocytes in colon samples of UC and Non UC patients. Leucocytes were isolated from colons of UC patients (n = 4) and Non UC patients (n = 5) undergoing colectomy. **a** Frequency of leucocyte; **b** Frequency of respective parent. *Bars* represent mean values and SD. A two-sided t-test and a significance level = 0.05 was used to compare groups

In order to verify the relevance of the identified markers in the affected colon, colon samples from UC patients undergoing colectomy were analyzed with regard to the presence of leucocytes and compared to unaffected areas of colon samples from cancer patients (Fig. 8). Leucocytes were isolated as described in “Methods” section and subjected to the same flow cytometric analysis as the leucocytes from blood samples (for complete data set see Additional file 1: Table S2, gating strategy Additional file 1: Fig. S3). Although frequencies of almost all cell types were increased in UC samples as compared to Non UC samples, a significant increase could only be determined for CD1a+ CD11b+ macrophages and NK T-cells both of which were significantly increased in the blood of UC patients. CD14+ CD1a+ monocytes just failed to reach significance (p = 0.08). CD11b expressing macrophages and monocytes and NK T-cells were the most abundant cell subpopulations in the inflamed colon.

## Discussion

Most studies to characterize inflammatory processes in UC are based on defining roles of single cell types or interleukins, chemokines or growth factors in the human disease and to validate their roles in an animal model. This approach has tremendously improved the understanding of inflammatory mechanism underlying the disease and to develop therapeutics targeting previously identified as crucial components. However, this approach does not take into consideration that inflammatory processes are rarely one dimensional and static but are better described as ‘mobilées’ in which there is crosstalk between static cells such as epithelial-, endothelial-muscle cells, fibrocytes and mobile inflammatory cells which can shift balances affecting the entire system in a time dependent manner. Therefore, we took a different approach.

First we analyzed frequencies of inflammatory cells in the blood and serum levels of certain factors of UC patients and compared them to Non UC patients. The selection of the subtypes of cells and the factors had a bias, as it was based on the hypothesis that part of the inflammation in UC resembled an uncontrolled wound healing process and therefore might be strongly influenced by macrophages and monocytes. Based on this immune-cell- and ELISA panel several significant differences in frequencies or concentrations between Non-UC donors and UC patients could be identified. These cells included cell of the adaptive immunity as plasma cells, cells of the innate immunity acting as mediators for the adaptive immunity as dendritic cells, CD11b+ macrophages and CD14+ monocytes and subtypes thereof as CD11b+ CD1a+ , CD14+ TSLPR+ , CD14+ CD1a+ , CD14+ CD163+, and cells of the innate immunity as NK T-, NK-cells and ILC2. In addition, HGF and TGFß1 which also have been previously described as elevated displayed a significant increase in serum levels [13, 43].

With the exception of NK T-cells age did not affect these cell types. Age and duration of disease resulted in decreased frequencies of naïve CD8+ and increase of frequencies of central memory CD8+ T-cells. This observation is in agreement with previous results, which also described a loss of naïve T-cells and an accumulation of experienced T- cells with age [40]. Thus, age might diminish the effect of the disease on these cell types. The observed increase of NK T-cells, however, might not be ascribed solely to the disease phenotype but also to age.

Gender did not affect expression levels of the analyzed immune cells. Since few of the patients were therapeutically naïve, one could not rule out that results were masked by a therapeutic effect on these cell types and factors. When UC patients were subdivided according to the respective therapy, the analysis revealed that TNFα-blockers (infliximab or adalizumab) had a profound effect on plasma cells, dendritic cells, TSLPR expressing CD11b+ macrophages and TSLPR expressing CD14+ monocytes. In this patient group, TARC and HGF serum levels were reduced, whereas TGFß1 levels were elevated. It is noteworthy that these patients—although most of them were in remission—did not regain the profile of a non UC subject but seem to represent a specific inflammatory condition. Opposing effects were observed in the glucocorticoid treated group. Cells of the adaptive immunity were not affected and in contrast to the group treated with TNFα-blockers frequencies of TSLPR expressing CD11b+ macrophages were elevated. Likewise, TARC and HGF serum levels were elevated as opposed to the group treated with TNFα-blockers. Mesalazine and immuno-suppressive drugs induced minor effects in this analysis. The group treated with immuno-suppressors was similar to the glucocorticoid treated group. As some of these patients were unresponsive to treatment, the observed effect might be due to the inflammatory condition and not the response to treatment. The cellular subtypes unaffected by all treatments and significantly different in UC patients and Non UC donors were CD1a expressing CD11b macrophages and monocytes.

CD1a expressing CD11b+ macrophages and CD14+ monocytes emerged as cell types significantly associated with UC. Both cell types differentiated between UC and Non UC donors with high discriminating potential as shown by an AUC value of 0.86 and 0.75, respectively. To our knowledge, CD1a expressing monocytes and macrophages have neither been associated with the gut nor with UC. The opposing effects of the treatment with glucocorticoids and TNFα-blockers suggested that these treatments might sustain different inflammatory conditions. This idea was supported by cluster- and correlation analysis. Unlike the previous analysis, cluster and correlation analysis can give information about individual inflammatory signatures and the inter dependencies of cells and factors. Both analyses suggested that two distinct inflammatory conditions prevail in UC patients: Treatment with TNFα-blockers induced a condition signified by CD14+ TSLPR+ monocytes which correlated positively with unswitched CD19+ B-cells and TGFß1 and periostin, indicating that this condition represents an inflammatory condition characterized by remodeling of the colon architecture (Table 4). The fact that most patients in this group responded to treatment and were considered as in remission further supported the idea. Of note, this condition does not reflect the homeostatic condition found in Non UC subjects, but more of an uncontrolled remodeling condition. These data might be consistent with studies in patients with Crohn’s disease which describe increased stenosis in response to treatment with infliximab. NK T-cells are considered the main source of IL-13 in UC [44] and our data suggest, that IL-13 might be the cytokine playing a key role in the remodeling condition.

The second inflammatory condition could be more characteristic of an acute inflammation signified by TSLPR+ CD11b+ macrophages that correlated positively with the SCCAI-Score, cells of the adaptive immunity, HGF and TARC. It is noteworthy that TSLPR+ CD11b+ and TSLPR+ CD14+ monocytes correlated negatively with each other, suggesting that treatment with TNFα-blockers clearly suppresses the acute condition while promoting the remodeling condition (Table 4). Our model might explain why anrukinzumab, an anti-IL-13 monoclonal antibody was found to have no effect on clinical activity score mucosal healing, rectal bleeding or clinical remission rates [45]. It might be that IL-13 may exert its activity in the remodeling condition by inducing tissue fibrosis [46] and not during the acute phase of the disease.

Furthermore, in Crohn’s disease it has been described that patients progress from inflammation to stenosis over a long period of time [47], suggesting that these two inflammatory conditions might also be found in Crohn’s patients. Finally, as an increased obstruction was observed in patients treated with infliximab, this drug is contraindicated in patients with stenosis. Our model might give also an explanation to these findings.

In this snapshot study, the dynamics of the disease had not been taken into consideration. Thus, we cannot conclude from our data that the inflammatory conditions relate to different stages such as relapse or remission. Longitudinal studies and studies with therapeutic naïve patients in remission might provide more insight into the dynamics of the disease.

In the intestine the inflammatory milieu in homeostasis and inflammation mainly depends on hematopoietic stem cell derived macrophages [48]. In the gut, macrophages are replenished from the blood and infiltrate the mucosa in case of inflammation. According to a new concept macrophages are considered accessory cell types which support their client cells—notably mucosal epithelial cells in the gut- with various functions. One major function is the clearance of apoptotic cells; however, in addition to this housekeeping function macrophages act as important sensors and can acquire functions on demand adapted to the inflammatory milieu ranging from pro-inflammatory to healing or regulatory [49]. This postmodern behavior challenges our view of the one-cell one-function approach in which the function of a cell can be identified by certain surface markers. Instead, we are confronted with a highly volatile cell population which shapes and is shaped by the inflammatory milieu. Thus, in the inflammatory condition of the gut epithelial cells might relay signals to macrophages and monocytes and depending of the presence or frequency of the TSLPR expressing macrophages or monocytes the outcome is tipped towards ‘acute’ or ‘remodeling’. Analysis of the second subtype of macrophages and monocytes, namely CD1a expressing CD11b+ macrophages, and CD14+ monocytes did not reveal a clear-cut functional segregation although CD1a+ macrophages correlate mainly with cell types and factors of the remodeling condition. Further studies have to be performed to analyze whether both cell types evoke different or similar responses.

The identification of potentially significant markers in the blood of UC patients is based on the assumption that the influx of inflammatory cells into the colonic mucosa is not a unidirectional route. Due to disrupted endothelial layers or an active transport mechanism, cell trafficking occurs to and from both compartments. If this assumption is correct one has to find the cells identified as relevant in the blood also in the colon of UC patient. Analysis of leucocytes from colons of UC identified monocytes, and CD1a+ CD11b+ macrophages and NK T-cells as significantly different in comparison to normal tissue derived from cancer patients undergoing colectomy. CD14+ CD1a+ monocytes just failed to reach significance. All three had been identified as significantly elevated markers in the blood of UC patients. Whether this observation reflects increased emigration to the colon or increased adherence to the colon has to be elucidated.

## Conclusions

Our results indicate that inflammation in UC is characterized by two main conditions: An ‘acute’ condition characterized by plasma cells, subtypes of CD11b+ macrophages, TARC and HGF and a ‘remodeling’ condition signified by NK T-cells, subtypes of CD14+ monocytes, TGFß1 and periostin. This study shows that profiling followed by correlation- and interrelation analysis of immune cells and serum factors leads to a better understanding of the ongoing inflammation. This analysis provides an explanation for the failure of anrukinzumab to reduce clinical symptoms and the observed increased stenosis in patients treated with infliximab. In addition, CD1a expressing monocytes and macrophages were identified as disease markers. We are confident that a holistic approach that will include a broader panel of immune cells and serum factors will result in defining subgroups of UC patients and will ultimately lead to more personalized therapies.

## Additional file

**Additional file 1: Figure. S1.** Gating strategy for isolated human PBMC.** Figure S2.** Age and duration of disease affects subtypes of leucocytes in UC patients.** Figure S3.** Gating strategy for human leucocytes isolated from human colon. **Table S1.** Data set for the flow cytometric analysis of PBMC and serum levels of HGF, TARC, TGFß1 and periostin.** Table S1A.** UC patients/Non UC donors.** Table S1B.** UC Patients treated with TNFα blockers/all other patients.** Table S1C.** UC Patients treated with glucocorticoids/all other patients. **Table S1D.** Patients treated with mesalazine/all other patients.** Table S1E.** Patients treated with immuno-suppressive drugs/all other patients.** Table S2.** Analysis of human Colon.

## References

[1] de Souza HS, Fiocchi C (2016). Immunopathogenesis of IBD: current state of the art. Nat Rev Gastroenterol Hepatol.

[2] Jovanovic K, Siebeck M, Gropp R (2014). The route to pathologies in chronic inflammatory diseases characterized by T helper type 2 immune cells. Clin Exp Immunol.

[3] Asokananthan N, Graham PT, Stewart DJ, Bakker AJ, Eidne KA, Thompson PJ, Stewart GA (2002). House dust mite allergens induce proinflammatory cytokines from respiratory epithelial cells: the cysteine protease allergen, Der p 1, activates protease-activated receptor (PAR)-2 and inactivates PAR-1. J Immunol.

[4] Vu AT, Baba T, Chen X, Le TA, Kinoshita H, Xie Y, Kamijo S, Hiramatsu K, Ikeda S, Ogawa H (2010). *Staphylococcus aureus* membrane and diacylated lipopeptide induce thymic stromal lymphopoietin in keratinocytes through the Toll-like receptor 2-Toll-like receptor 6 pathway. J Allergy Clin Immunol..

[5] Le TA, Takai T, Vu AT, Kinoshita H, Chen X, Ikeda S, Ogawa H, Okumura K (2011). Flagellin induces the expression of thymic stromal lymphopoietin in human keratinocytes via toll-like receptor 5. Int Arch Allergy Immunol.

[6] Li DQ, Zhang L, Pflugfelder SC, De Paiva CS, Zhang X, Zhao G, Zheng X, Su Z, Qu Y (2011). Short ragweed pollen triggers allergic inflammation through Toll-like receptor 4-dependent thymic stromal lymphopoietin/OX40 ligand/OX40 signaling pathways. J Allergy Clin Immunol.

[7] Xie Y, Takai T, Chen X, Okumura K, Ogawa H (2012). Long TSLP transcript expression and release of TSLP induced by TLR ligands and cytokines in human keratinocytes. J Dermatol Sci.

[8] Yamada T, Saito H, Kimura Y, Kubo S, Sakashita M, Susuki D, Ito Y, Ogi K, Imoto Y, Fujieda S (2012). CpG-DNA suppresses poly(I:C)-induced TSLP production in human laryngeal arytenoid fibroblasts. Cytokine.

[9] Takai T, Chen X, Xie Y, Vu AT, Le TA, Kinoshita H, Kawasaki J, Kamijo S, Hara M, Ushio H (2014). TSLP expression induced via Toll-like receptor pathways in human keratinocytes. Methods Enzymol.

[10] Scallon BJ, Moore MA, Trinh H, Knight DM, Ghrayeb J (1995). Chimeric anti-TNF-alpha monoclonal antibody cA2 binds recombinant transmembrane TNF-alpha and activates immune effector functions. Cytokine.

[11] Mosser DM, Edwards JP (2008). Exploring the full spectrum of macrophage activation. Nat Rev Immunol.

[12] Ferrante CJ, Leibovich SJ (2012). Regulation of macrophage polarization and wound healing. Adv Wound Care (New Rochelle).

[13] Christophi GP, Rong R, Holtzapple PG, Massa PT, Landas SK (2012). Immune markers and differential signaling networks in ulcerative colitis and Crohn’s disease. Inflamm Bowel Dis.

[14] Srivastava M, Zurakowski D, Cheifetz P, Leichtner A, Bousvaros A (2001). Elevated serum hepatocyte growth factor in children and young adults with inflammatory bowel disease. J Pediatr Gastroenterol Nutr.

[15] Molnarfi N, Benkhoucha M, Funakoshi H, Nakamura T, Lalive PH (2014). Hepatocyte growth factor: a regulator of inflammation and autoimmunity. Autoimmun Rev.

[16] Kajdaniuk D, Marek B, Borgiel-Marek H, Kos-Kudla B (2013). Transforming growth factor beta1 (TGFbeta1) in physiology and pathology. Endokrynol Pol.

[17] Werner S, Grose R (2003). Regulation of wound healing by growth factors and cytokines. Physiol Rev.

[18] Saeki H, Tamaki K (2006). Thymus and activation regulated chemokine (TARC)/CCL17 and skin diseases. J Dermatol Sci.

[19] Gear AR, Camerini D (2003). Platelet chemokines and chemokine receptors: linking hemostasis, inflammation, and host defense. Microcirculation.

[20] Parulekar AD, Atik MA, Hanania NA (2014). Periostin, a novel biomarker of TH2-driven asthma. Curr Opin Pulm Med.

[21] Sidhu SS, Yuan S, Innes AL, Kerr S, Woodruff PG, Hou L, Muller SJ, Fahy JV (2010). Roles of epithelial cell-derived periostin in TGF-beta activation, collagen production, and collagen gel elasticity in asthma. Proc Natl Acad Sci USA.

[22] de Jong A, Pena-Cruz V, Cheng TY, Clark RA, Van Rhijn I, Moody DB (2010). CD1a-autoreactive T cells are a normal component of the human alphabeta T cell repertoire. Nat Immunol.

[23] de Jong A, Cheng TY, Huang S, Gras S, Birkinshaw RW, Kasmar AG, Van Rhijn I, Pena-Cruz V, Ruan DT, Altman JD (2014). CD1a-autoreactive T cells recognize natural skin oils that function as headless antigens. Nat Immunol.

[24] Basu R, O’Quinn DB, Silberger DJ, Schoeb TR, Fouser L, Ouyang W, Hatton RD, Weaver CT (2012). Th22 cells are an important source of IL-22 for host protection against enteropathogenic bacteria. Immunity.

[25] Brand S, Beigel F, Olszak T, Zitzmann K, Eichhorst ST, Otte JM, Diepolder H, Marquardt A, Jagla W, Popp A (2006). IL-22 is increased in active Crohn’s disease and promotes proinflammatory gene expression and intestinal epithelial cell migration. Am J Physiol Gastrointest Liver Physiol.

[26] Sabat R, Ouyang W, Wolk K (2014). Therapeutic opportunities of the IL-22-IL-22R1 system. Nat Rev Drug Discov.

[27] Bourgeois EA, Subramaniam S, Cheng TY, De Jong A, Layre E, Ly D, Salimi M, Legaspi A, Modlin RL, Salio M (2015). Bee venom processes human skin lipids for presentation by CD1a. J Exp Med.

[28] Thasler WE, Weiss TS, Schillhorn K, Stoll PT, Irrgang B, Jauch KW (2003). charitable state-controlled foundation human tissue and cell research: ethic and legal aspects in the supply of surgically removed human tissue for research in the academic and commercial sector in Germany. Cell Tissue Bank.

[29] Uronen-Hansson H, Persson E, Nilsson P, Agace W. Isolation of cells from human intestinal tissue. Bio-protocol. 2014;4:e1092. http://wwwbio-protocol.org/e1092.

[30] Sanz I, Wei C, Lee FE, Anolik J (2008). Phenotypic and functional heterogeneity of human memory B cells. Semin Immunol.

[31] Lehmann J, Huehn J, de la Rosa M, Maszyna F, Kretschmer U, Krenn V, Brunner M, Scheffold A, Hamann A (2002). Expression of the integrin alpha Ebeta 7 identifies unique subsets of CD25+ as well as CD25- regulatory T cells. Proc Natl Acad Sci USA.

[32] Zaunders JJ, Munier ML, Seddiki N, Pett S, Ip S, Bailey M, Xu Y, Brown K, Dyer WB, Kim M (2009). High levels of human antigen-specific CD4+ T cells in peripheral blood revealed by stimulated coexpression of CD25 and CD134 (OX40). J Immunol.

[33] Guilliams M, Ginhoux F, Jakubzick C, Naik SH, Onai N, Schraml BU, Segura E, Tussiwand R, Yona S (2014). Dendritic cells, monocytes and macrophages: a unified nomenclature based on ontogeny. Nat Rev Immunol.

[34] Murray PJ, Wynn TA (2011). Protective and pathogenic functions of macrophage subsets. Nat Rev Immunol.

[35] Orme J, Mohan C (2012). Macrophage subpopulations in systemic lupus erythematosus. Discov Med.

[36] Gordon S, Taylor PR (2005). Monocyte and macrophage heterogeneity. Nat Rev Immunol.

[37] Verreck FA, de Boer T, Langenberg DM, van der Zanden L, Ottenhoff TH (2006). Phenotypic and functional profiling of human proinflammatory type-1 and anti-inflammatory type-2 macrophages in response to microbial antigens and IFN-gamma- and CD40L-mediated costimulation. J Leukoc Biol.

[38] Thanapati S, Das R, Tripathy AS (2015). Phenotypic and functional analyses of NK and NKT-like populations during the early stages of chikungunya infection. Front Microbiol.

[39] Ljunggren HG, Karre K (1990). In search of the ‘missing self’: MHC molecules and NK cell recognition. Immunol Today.

[40] Vescovini R, Fagnoni FF, Telera AR, Bucci L, Pedrazzoni M, Magalini F, Stella A, Pasin F, Medici MC, Calderaro A (2014). Naive and memory CD8 T cell pool homeostasis in advanced aging: impact of age and of antigen-specific responses to cytomegalovirus. Age (Dordr).

[41] Jowett SL, Seal CJ, Phillips E, Gregory W, Barton JR, Welfare MR (2003). Defining relapse of ulcerative colitis using a symptom-based activity index. Scand J Gastroenterol.

[42] Walmsley RS, Ayres RC, Pounder RE, Allan RN (1998). A simple clinical colitis activity index. Gut.

[43] Feng JS, Yang Z, Zhu YZ, Liu Z, Guo CC, Zheng XB (2014). Serum IL-17 and IL-6 increased accompany with TGF-beta and IL-13 respectively in ulcerative colitis patients. Int J Clin Exp Med.

[44] Fuss IJ, Heller F, Boirivant M, Leon F, Yoshida M, Fichtner-Feigl S, Yang Z, Exley M, Kitani A, Blumberg RS (2004). Nonclassical CD1d-restricted NK T cells that produce IL-13 characterize an atypical Th2 response in ulcerative colitis. J Clin Invest.

[45] Reinisch W, Panes J, Khurana S, Toth G, Hua F, Comer GM, Hinz M, Page K, O’Toole M, Moorehead TM (2015). Anrukinzumab, an anti-interleukin 13 monoclonal antibody, in active UC: efficacy and safety from a phase IIa randomised multicentre study. Gut.

[46] Fichtner-Feigl S, Fuss IJ, Young CA, Watanabe T, Geissler EK, Schlitt HJ, Kitani A, Strober W (2007). Induction of IL-13 triggers TGF-beta1-dependent tissue fibrosis in chronic 2,4,6-trinitrobenzene sulfonic acid colitis. J Immunol.

[47] Vermeire S, Van Assche G, Rutgeerts P (2007). Review article: altering the natural history of Crohn’s disease—evidence for and against current therapies. Aliment Pharmacol Ther.

[48] Bain CC, Bravo-Blas A, Scott CL, Gomez Perdiguero E, Geissmann F, Henri S, Malissen B, Osborne LC, Artis D, Mowat AM (2014). Constant replenishment from circulating monocytes maintains the macrophage pool in the intestine of adult mice. Nat Immunol.

[49] Okabe Y, Medzhitov R (2015). Tissue biology perspective on macrophages. Nat Immunol.

