# Supplementary material for: Immunological profiling of patients with ulcerative colitis leads to identification of two inflammatory conditions and CD1a as a disease marker
Source: J Transl Med. 2016 Nov 3;14:310. doi: 10.1186/s12967-016-1048-9 (PMC5094062; doi:10.1186/s12967-016-1048-9)
Supplement: Supplementary file 1 — Additional file 1: Figure. S1. Gating strategy for isolated human PBMC. Figure S2. Age and duration of disease affects subtypes of leucocytes in UC patients. Figure S3. Gating strategy for human leucocytes isolated from human colon. Table S1. Data set for the flow cytometric analysis of PBMC and serum levels of HGF, TARC, TGFß1 and periostin. Table S1A. UC patients/Non UC donors. Table S1B. UC Patients treated with TNFα blockers/all other patients. Table S1C. UC Patients treated with glucocorticoids/all other patients. Table S1D. Patients treated with mesalazine/all other patients. Table S1E. Patients treated with immuno-suppressive drugs/all other patients. Table S2. Analysis of human Colon. [file 12967_2016_1048_MOESM1_ESM.docx]

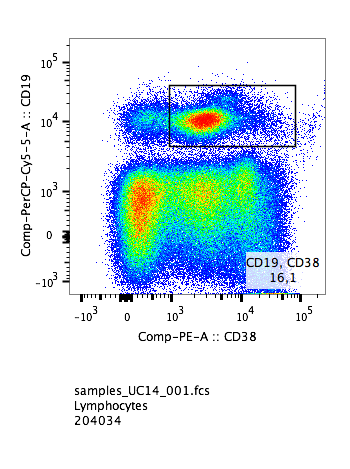

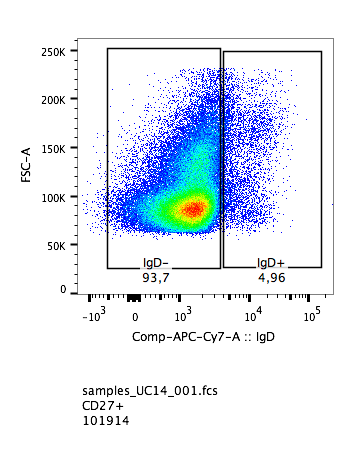

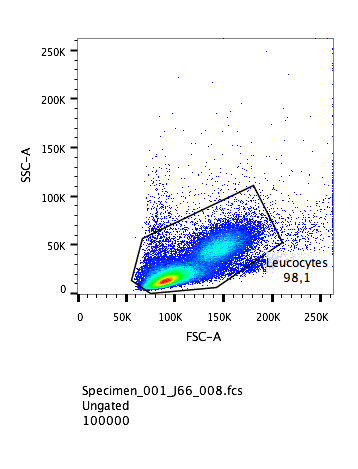

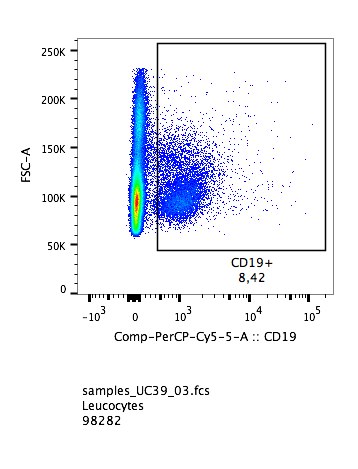

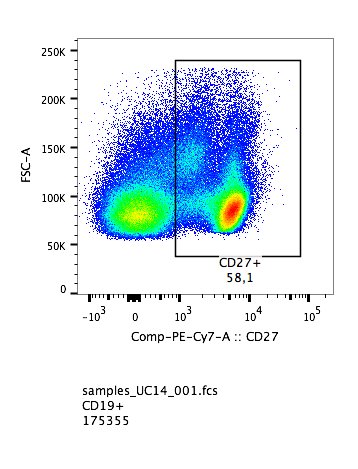


**Gate 1**

**CD19+ CD38+**

**CD19+**

**CD19+ CD27+**

**CD19+ CD27+ IgD±**


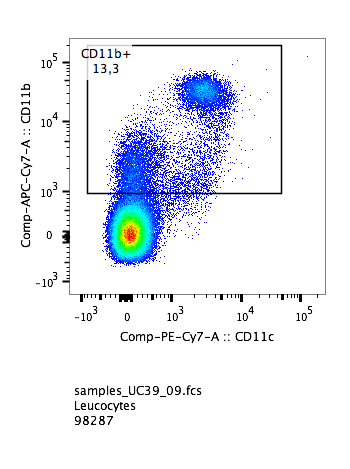

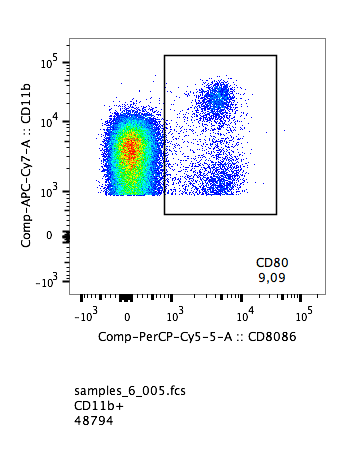

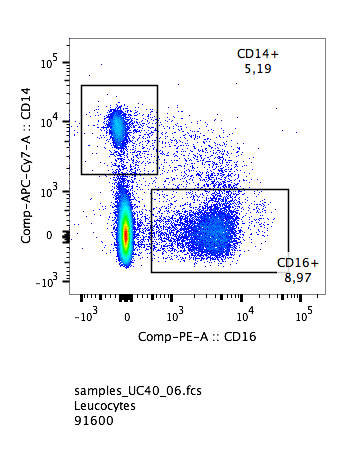

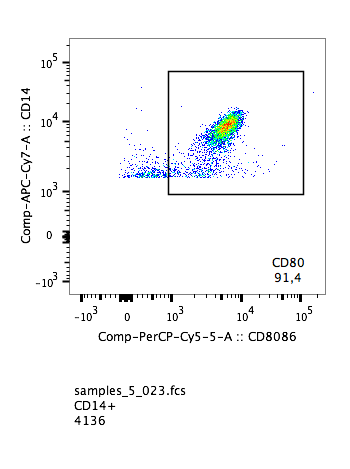

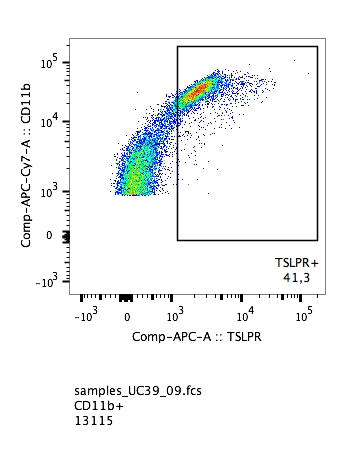

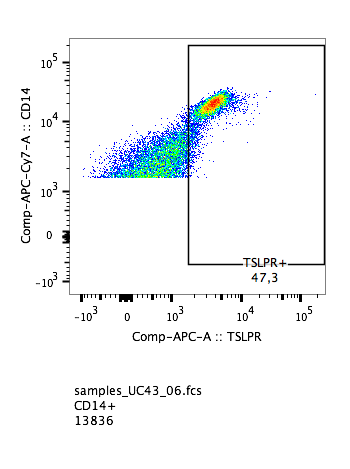


**Gate 1**

**CD11b+**

**CD11b+ CD1a+**

**CD11b+ TSLPR+**

**CD11b+ CD80+**

**Gate 1**

**CD14+ /CD16+**

**CD14+ CD1a+**

**CD14+ TSLPR+**

**CD14+ CD80+**


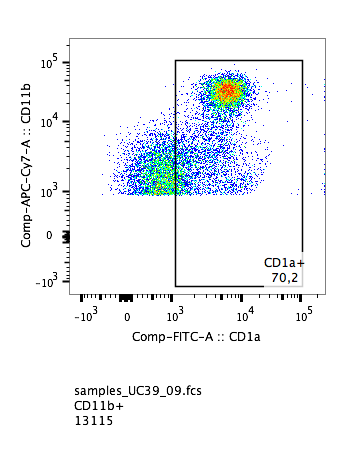

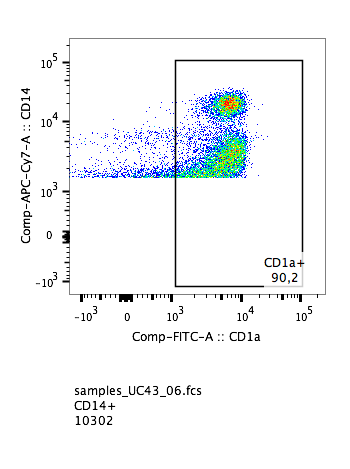

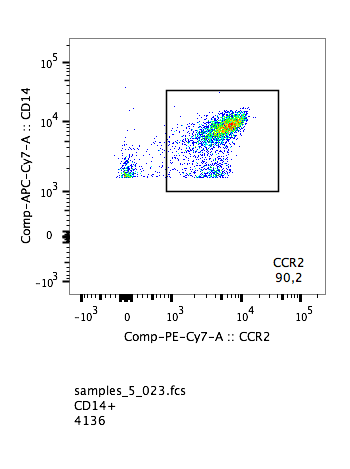


**CD14+ CCR2+**


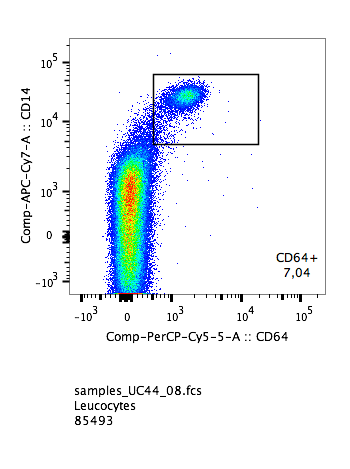

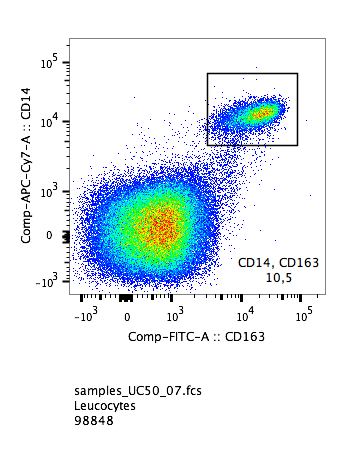


**CD14+ CD64+**

**CD14+ CD163+**

**Gate 1**


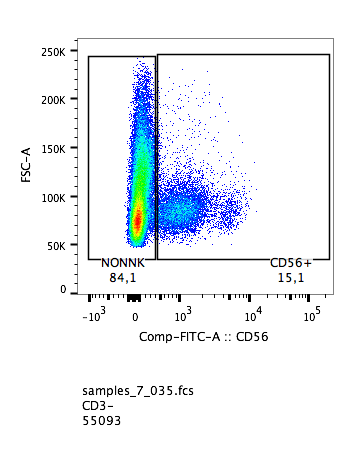

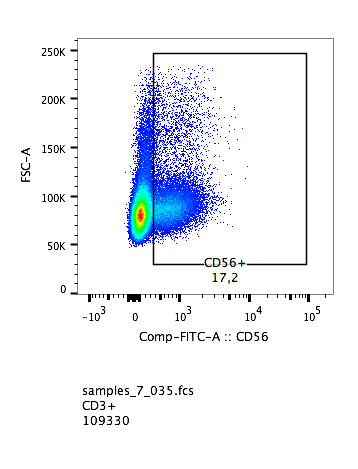

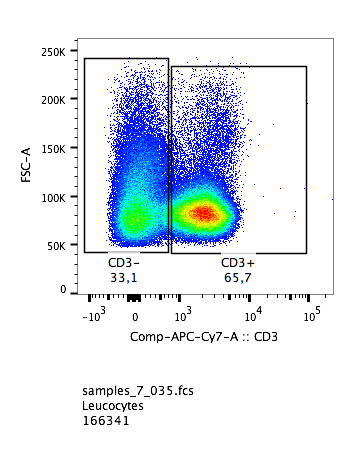


**CD3±**

**CD3- CD56±**

**CD3+ CD56±**

**Gate 1**

**Fig. S1. Gating stategy for isolated human PBMC.**


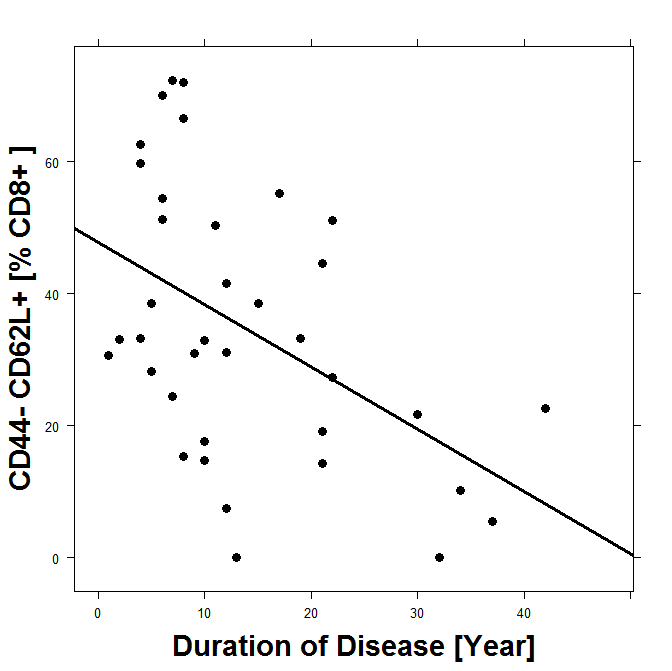

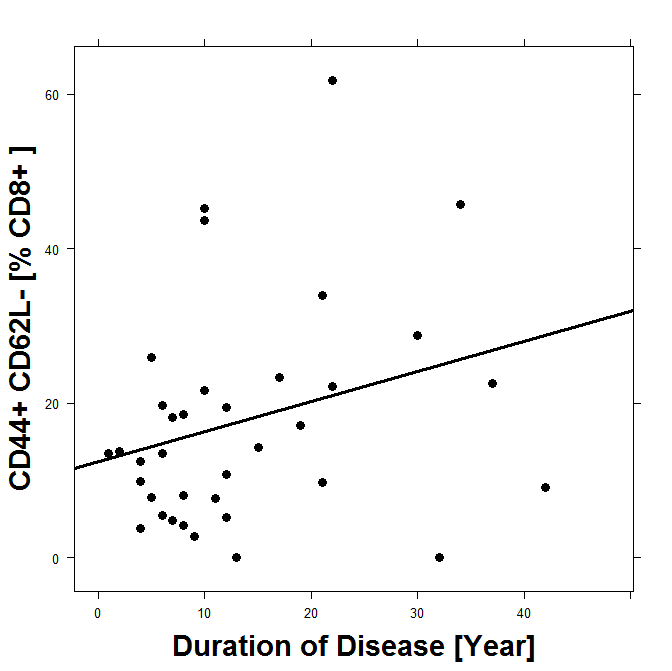

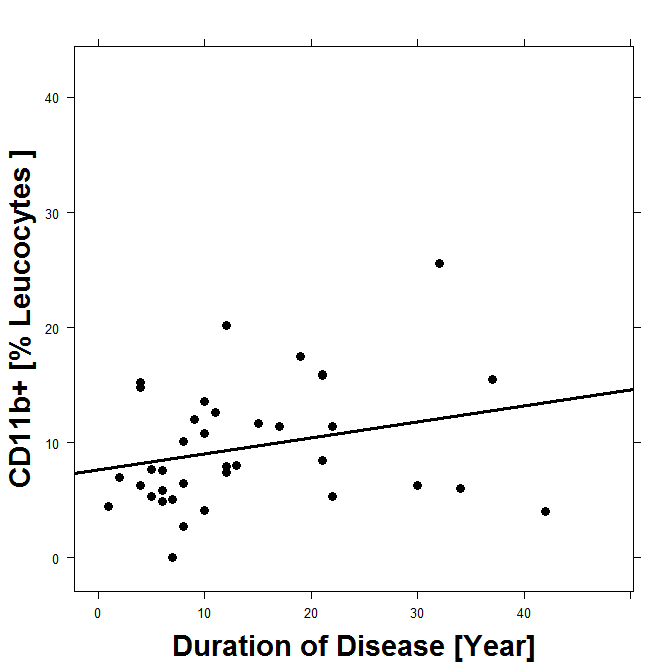

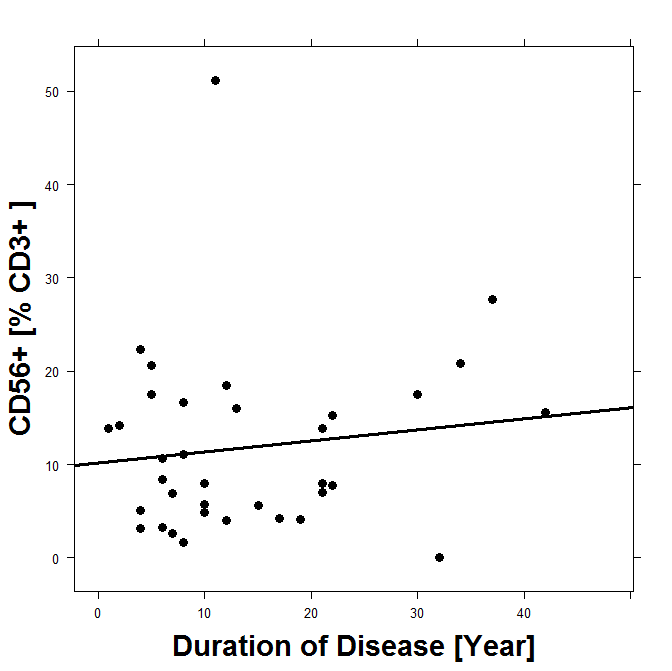

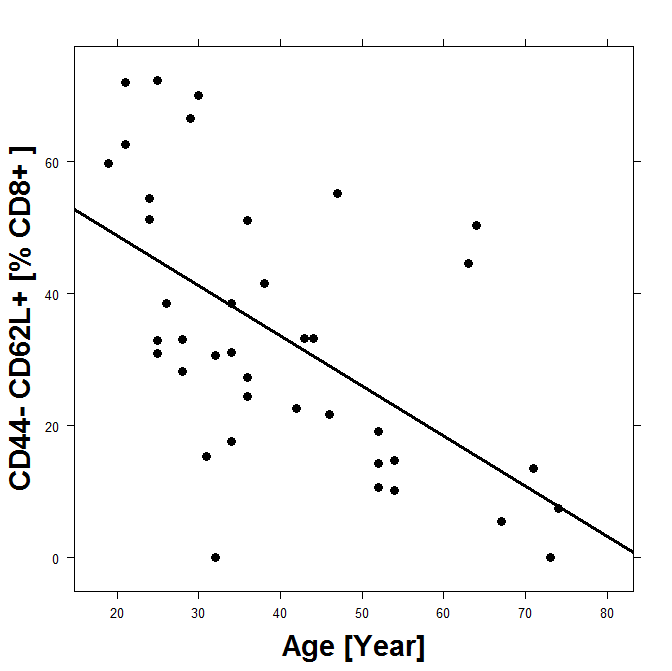

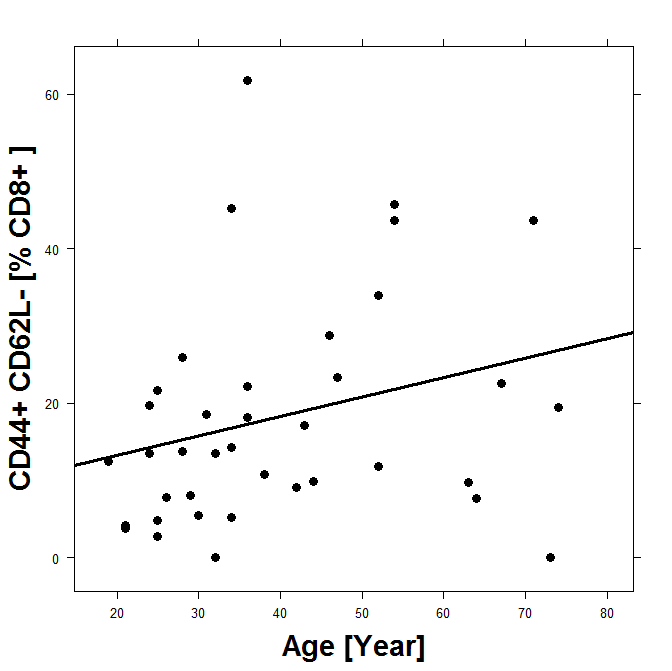


**rho= -0.61**

**p= 1.4e-05**

**rho= 0.35**

**p= 0.01**

**rho= -0.48**

**p= 0.001**

**rho= 0.3**

**p= 0.03**

**rho= 0.29**

**p= 0.04**


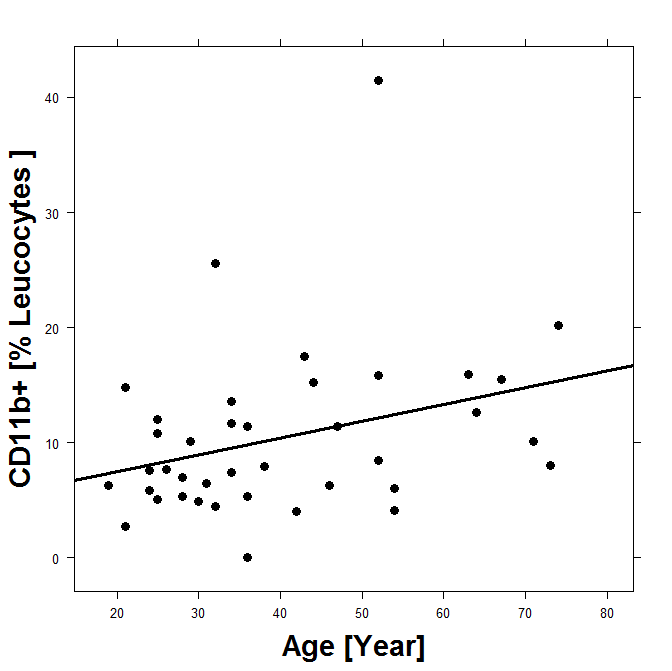


**rho= 0.35**

**p= 0.01**


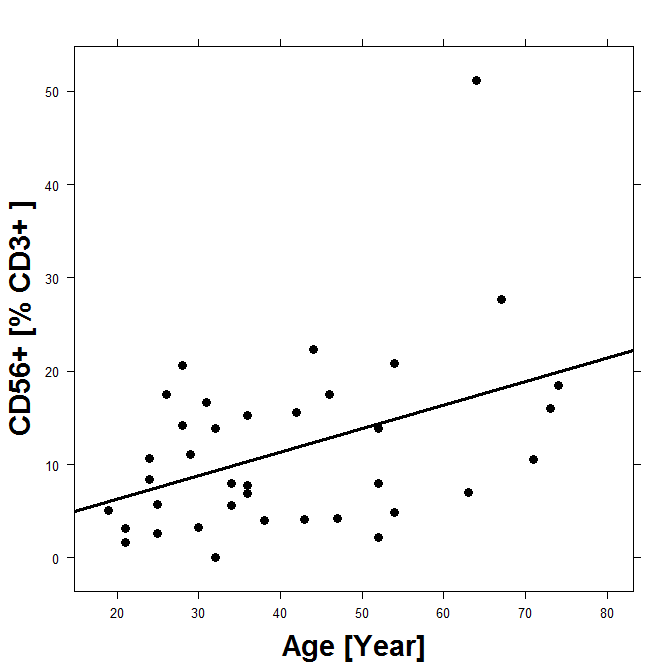


**rho= 0.36**

**p= 0.01**

**Fig. S2. Age and duration of disease affects subtypes of leucocytes in UC patients.** Correlation analysis depicted as xyplots. Spearman rank-order correlation coefficients rho and p-values. Sample size: CD3+ 56+NK T-cells n=35, CD8+ CD44- CD62L+ -, CD8+ CD44+ CD62L, CD11b+ macrophages n=37.


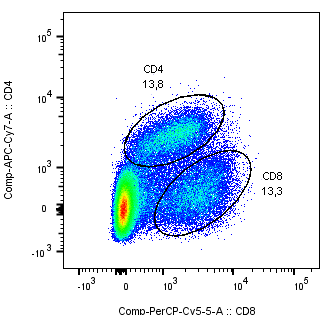

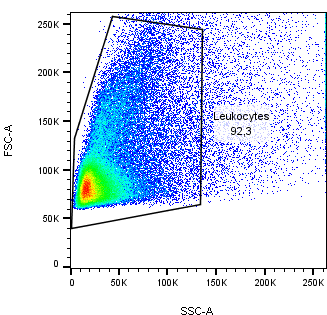

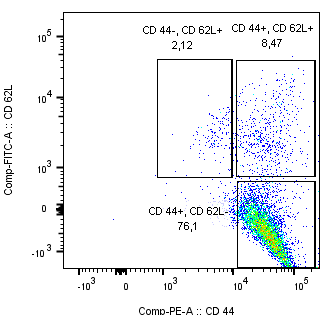

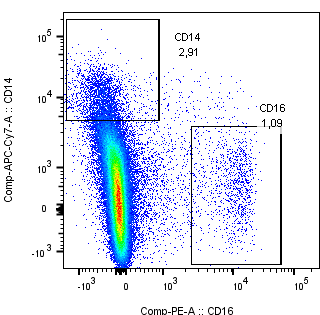

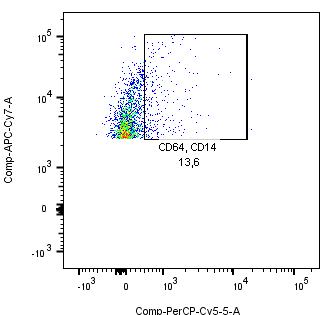

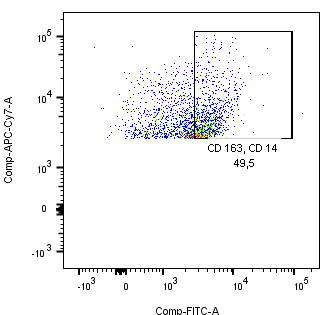

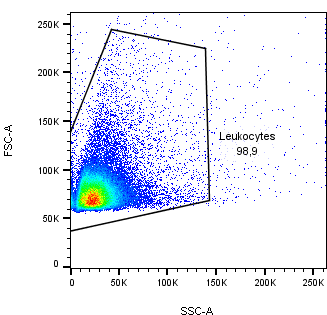

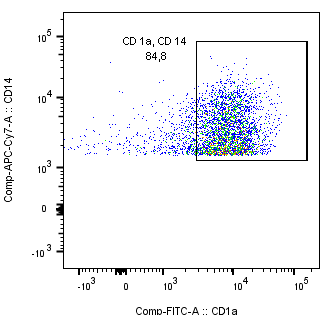

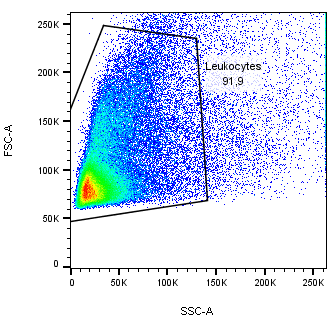

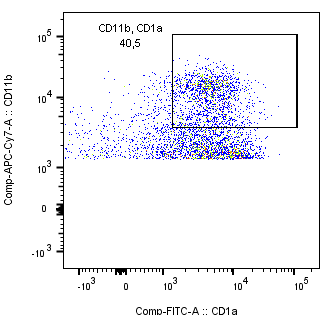

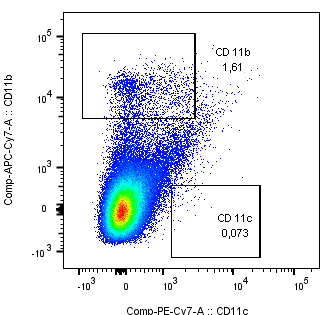

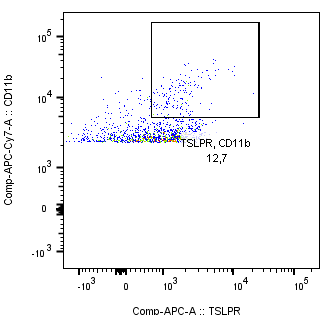

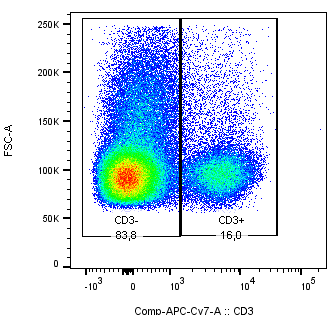

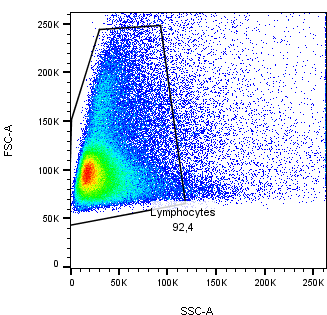

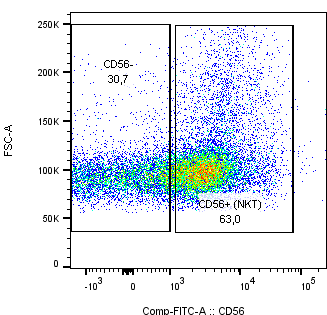


**CD4+ CD44± CD62L±**

**CD11b+ CD11c+**

**CD11b+ CD1a+**

**CD11b+ TSLPR+**

**CD8+ CD8+**

**CD14+ CD16+**

**CD14+ CD64**

**CD14+ CD163**

**CD14+ CD1a**

**CD3±**

**CD3+ CD56+**


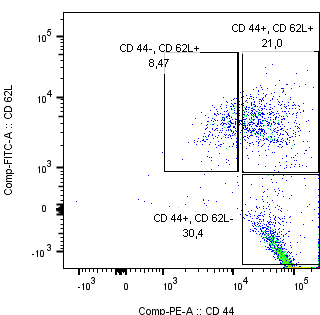


**CD8+ CD44± CD62L±**

**Fig. S3. Gating strategy for human leucocytes isolated from human colon.**

**Table S1 Data set for the flow cytometric analysis of PBMC and serum levels of HGF, TARC, TGFß1 and periostin.**

**Table S1A UC patients / Non UC donors**

|  | **Mean** | | **SD** | | **IQR** | | **n** | | **Δ** | **p-value** | **95% CI** |
| --- | --- | --- | --- | --- | --- | --- | --- | --- | --- | --- | --- |
| **Leukocytes [FoP]** | **Non UC** | **UC** | **Non UC** | **UC** | **Non UC** | **UC** | **Non UC** | **UC** |  |  |  |
| **CD19+** | **25,56** | **16,76** | **17,37** | **20,34** | **26,84** | **7,63** | **31** | **40** | **-8,80** | **0,05** | **-0,1 - 17,7** |
| **CD19+CD27+ IgD+** | **24,22** | **18,15** | **20,72** | **18,01** | **28,69** | **20,80** | **15** | **40** | **-6,07** |  |  |
| **CD19+ CD27+ IgD-** | **67,01** | **77,58** | **27,69** | **22,85** | **35,95** | **25,52** | **15** | **40** | **10,57** |  |  |
| **CD19+ CD38+** | **60,70** | **23,60** | **32,25** | **29,26** | **52,20** | **40,36** | **31** | **40** | **-37,10** | **5E-03** | **23,4 - 52,5** |
| **CD4+** | **32,72** | **24,16** | **12,62** | **12,56** | **16,90** | **16,24** | **30** | **40** | **-8,56** | **0,006** | **2,7 - 14,9** |
| **CD4+ CD25+** | **12,17** | **5,01** | **6,83** | **6,03** | **7,98** | **1,17** | **30** | **40** | **-7,16** | **3E-05** | **4,1 - 10,4** |
| **CD8+** | **16,54** | **16,35** | **7,77** | **10,13** | **7,20** | **13,15** | **30** | **40** | **-0,19** |  |  |
| **CD11b+** | **17,18** | **10,87** | **10,92** | **7,68** | **8,60** | **7,91** | **31** | **40** | **-6,31** | **0.005** | **2,1 - 11,2** |
| **CD11b+ TSLPR+** | **15,14** | **18,87** | **14,36** | **23,99** | **13,54** | **16,78** | **15** | **40** | **3,73** |  |  |
| **CD11b+ CD1a+** | **5,07** | **22,80** | **17,97** | **23,33** | **0,87** | **27,39** | **31** | **40** | **17,73** | **0.0005** | **-27,9 - -8,3** |
| **CD11b+ CD80/86+** | **11,30** | **10,33** | **17,67** | **17,93** | **14,86** | **5,98** | **31** | **39** | **-0,97** |  |  |
| **CD11c+** | **1,43** | **0,87** | **0,67** | **0,68** | **0,76** | **0,83** | **31** | **40** | **-0,56** | **0.0002** | **0,3 - 0,9** |
| **CD14+** | **6,00** | **5,86** | **4,18** | **6,37** | **6,73** | **6,53** | **30** | **40** | **-0,14** |  |  |
| **CD14+ CCR2+** | **61,67** | **77,25** | **42,61** | **24,44** | **90,76** | **20,55** | **30** | **40** | **15,58** |  |  |
| **CD14+ CD80/86+** | **48,01** | **37,32** | **47,13** | **35,31** | **94,51** | **72,67** | **30** | **40** | **-10,69** |  |  |
| **CD14+ TSLPR+** | **3,16** | **6,69** | **4,63** | **8,54** | **1,50** | **6,91** | **15** | **40** | **3,53** | **0.03** | **-8,4 - -0,5** |
| **CD14+ CD1a+** | **59,36** | **79,71** | **22,41** | **22,80** | **30,6** | **7,40** | **9** | **27** | **20,35** | **0.02** | **-40,8 - -4,5** |
| **CD14+ CD64+** | **10,97** | **1,10** | **6,69** | **1,34** | **7,38** | **0,55** | **4** | **25** | **-9,87** | **0,06** |  |
| **CD14+ CD163+** | **6,08** | **2,54** | **4,80** | **2,31** | **7,73** | **2,68** | **10** | **26** | **-3,54** | **0.05** | **6,0 - 2,5** |
| **CD3- CD56+** | **13,27** | **12,62** | **12,56** | **12,57** | **9,12** | **12,30** | **13** | **38** | **-0,65** |  |  |
| **CD3+ CD56+** | **5,64** | **11,99** | **3,64** | **9,96** | **2,53** | **11,52** | **13** | **38** | **6,35** | **0.001** | **-10,3 - -2,6** |
| **CD3+ CD56+ KIR+** | **61,00** | **27,12** | **43,49** | **20,09** | **88,50** | **30,42** | **13** | **38** | **-33,88** | **0.01** | **7,7 - 61,5** |
| **ILC2** | **2,05** | **1,33** | **1,30** | **0,87** | **1,20** | **0,93** | **13** | **38** | **-0,72** | **0.08** |  |
| **Factors [ng / ml]** |  |  |  |  |  |  |  |  |  |  |  |
| **TARC** | **5.222,43** | **7.009,40** | **2.935** | **6.169** | **4.020** | **9.160** | **26** | **41** | **1786,97** |  |  |
| **HGF** | **868,44** | **2.278,55** | **876** | **2.191** | **463** | **2.743** | **27** | **39** | **1410,11** | **0.0006** | **-2190,8 - -629,3** |
| **TGFß1** | **23534,53** | **40298,48** | **14004,41** | **30694,27** | **15958,18** | **49604,05** | **23** | **42** | **16763,95** | **0.003** | **23534,5 - 38143,0** |
| **Periostin** | **74.066,78** | **60.635,67** | **39631,19** | **38621,69** | **31787,63** | **38485,27** | **17** | **36** | **-13431,11** |  |  |

**Table S1B. UC Patients treated with TNFα blockers / all other patients**

|  | **Mean** | | **SD** | | **IQR** | | **n** | | **Δ** | **p-value** | **95% CI** |
| --- | --- | --- | --- | --- | --- | --- | --- | --- | --- | --- | --- |
| **Leukocytes [FoP]** | **TNFa-blocker** | **other** | **TNFa-blocker** | **other** | **TNFa-blocker** | **other** | **TNFa-blocker** | **No TNFa-blocker** |  |  |  |
| **CD19+** | **11,80** | **22,58** | **15,50** | **23,14** | **4,63** | **25,22** | **21** | **20** | **-10,78** |  |  |
| **CD19+ CD27+ IgD+** | **20,46** | **14,96** | **17,63** | **18,23** | **18,27** | **19,75** | **21** | **20** | **5,50** |  |  |
| **CD19+ CD27+ IgD-** | **79,31** | **75,85** | **12,06** | **30,11** | **20,85** | **28,17** | **20** | **20** | **3,47** |  |  |
| **CD19+ 38+** | **9,87** | **39,69** | **19,02** | **30,64** | **1,83** | **47,04** | **21** | **20** | **-29,82** | **8 E-4** | **-45,8 - -13,2** |
| **CD4+** | **24,94** | **22,94** | **11,93** | **13,72** | **16,70** | **18,50** | **21** | **20** | **2,00** |  |  |
| **CD4+ CD25+** | **3,92** | **6,12** | **1,61** | **8,34** | **0,98** | **2,38** | **21** | **20** | **-2,20** |  |  |
| **CD8+** | **13,97** | **18,72** | **8,49** | **11,24** | **12,02** | **13,70** | **21** | **20** | **-4,75** |  |  |
| **CD11b+** | **8,81** | **12,93** | **4,01** | **9,80** | **5,27** | **10,94** | **21** | **20** | **-4,12** |  |  |
| **CD11b+ TSLPR+** | **10,96** | **26,32** | **13,18** | **29,77** | **9,99** | **25,81** | **21** | **20** | **-15,36** | **0.04** | **-30,26 - -0,44** |
| **CD11b+ CD1a+** | **24,85** | **20,75** | **23,19** | **23,88** | **24,47** | **26,59** | **21** | **20** | **4,10** |  |  |
| **CD11b+ CD80+** | **5,37** | **18,23** | **3,66** | **26,54** | **3,35** | **14,14** | **21** | **20** | **-12,86** | **0.04** | **-25,3 - -0,35** |
| **CD11c+** | **0,64** | **1,10** | **0,46** | **0,80** | **0,57** | **1,03** | **21** | **20** | **-0,46** |  |  |
| **CD14+** | **5,43** | **6,29** | **4,48** | **7,90** | **6,19** | **6,88** | **21** | **20** | **-0,86** |  |  |
| **CD14+ CCR2+** | **74,36** | **80,14** | **25,84** | **23,25** | **20,32** | **17,15** | **21** | **20** | **-5,78** |  |  |
| **CD14+ CD80/86+** | **42,03** | **34,00** | **36,07** | **36,22** | **76,47** | **61,17** | **21** | **20** | **8,03** |  |  |
| **CD14+ TSLPR+** | **10,74** | **4,00** | **11,93** | **5,06** | **5,85** | **5,66** | **21** | **20** | **6,74** | **0.03** | **0,8 - 12,4** |
| **CD14+ CD1a+** | **84,95** | **78,04** | **11,34** | **29,60** | **6,30** | **7,80** | **18** | **9** | **6,91** |  |  |
| **CD14+ CD64+** | **1,09** | **1,23** | **1,58** | **0,64** | **0,18** | **1,01** | **17** | **8** | **-0,14** |  |  |
| **CD14+ CD163+** | **2,68** | **2,53** | **2,49** | **2,09** | **2,59** | **3,11** | **18** | **9** | **0,15** |  |  |
| **CD3- CD56+** | **12,99** | **13,57** | **7,94** | **10,51** | **8,73** | **11,69** | **21** | **18** | **-0,58** |  |  |
| **CD3+ CD56+** | **11,32** | **12,73** | **8,06** | **11,93** | **10,11** | **12,82** | **21** | **18** | **-1,41** |  |  |
| **ILC2** | **1,46** | **1,18** | **0,97** | **0,77** | **0,94** | **0,99** | **20** | **18** | **0,28** |  |  |
| **Factors [ng / ml]** |  |  |  |  |  |  |  |  |  |  |  |
| **TARC** | **3.257,75** | **9.945,47** | **4.064** | **5.993** | **3.225** | **9.095** | **18** | **23** | **-6687,72** | **0.0001** | **3257,7 - 9945,4** |
| **HGF** | **1.084,54** | **3.109,16** | **1.393** | **2.283** | **545** | **3.062** | **16** | **23** | **2024,62** | **0.001** | **-3220,3 - -828,84** |
| **TGFß1** | **52.946,12** | **30.812,75** | **20043,09** | **34109,48** | **15021,03** | **41763,36** | **18** | **24** | **-22133,37** | **0.01** | **52946,1 - 30812,8** |
| **Periostin** | **72.395,26** | **51.229,01** | **47663,26** | **27287,68** | **27740,2** | **37911,48** | **16** | **20** | **-21166,25** |  |  |

**Table S1C UC Patients treated with glucocorticoids/ all other patients**

|  | **Mean** | | **SD** | | **IQR** | | **n** | | **Δ** | **p-value** | **95% CI** |
| --- | --- | --- | --- | --- | --- | --- | --- | --- | --- | --- | --- |
| **Leukocytes [FoP]** | **Glucocorticoids** | **other** | **Glucocorticoids** | **other** | **Glucocorticoids** | **other** | **Glucocorticoids** | **No Glucocorticoids** |  |  |  |
| CD19+ | 34 | 10,35 | 27,81 | 11,36 | 50,9 | 6,65 | 11 | 30 | 24,07 | 0.01 | 5,1 - 43,0 |
| CD19+ CD27+ IgD+ | 7,77 | 21,44 | 11,84 | 18,54 | 1,9 | 21,32 | 11 | 30 | -13,67 | 0,009 | -23,74 - 3,58 |
| CD19+ CD27+ IgD- | 90,32 | 71,67 | 12,32 | 23,77 | 1,95 | 21,32 | 11 | 30 | 18,65 | 0.002 | 7,0 - 30,27 |
| CD19+ 38+ | 35,09 | 20,34 | 16,82 | 31,86 | 14,4 | 38,53 | 11 | 30 | 14,75 | 0,06 |  |
| CD4+ | 22,15 | 24,86 | 11,03 | 13,2 | 11,6 | 15,7 | 11 | 29 | -2,71 |  |  |
| CD4+ CD25+ | 4,52 | 5,2 | 1,51 | 7,05 | 0,83 | 1,07 | 11 | 30 | -0,68 |  |  |
| CD4+ CD294+ | 2,24 | 1,39 | 1,94 | 1,35 | 1,09 | 0,66 | 11 | 29 | 0,85 |  |  |
| CD8+ | 17,29 | 15,99 | 7,69 | 11,02 | 11,72 | 13,56 | 11 | 30 | 1,30 |  |  |
| CD11b+ | 12,72 | 9,87 | 10,52 | 5,57 | 8,15 | 6,66 | 11 | 30 | 2,85 |  |  |
| CD11b+ TSLPR+ | 35,92 | 12 | 31,07 | 17,11 | 31,7 | 8,42 | 11 | 30 | 23,92 | 0.03 | 2,4 - 45,3 |
| CD11b+ CD1a+ | 13,63 | 26,28 | 22,76 | 22,96 | 13,77 | 16,3 | 11 | 30 | -12,65 |  |  |
| CD11b+ CD80+ | 11,92 | 11,54 | 22,72 | 18,74 | 5,19 | 4,32 | 11 | 30 | 0,38 |  |  |
| CD11c+ | 0,81 | 0,89 | 0,72 | 0,68 | 0,96 | 0,67 | 11 | 30 | -0,08 |  |  |
| CD14+ | 6,13 | 5,76 | 9,88 | 4,64 | 4,54 | 7,15 | 11 | 30 | 0,37 |  |  |
| CD14+ CCR2+ | 85,77 | 74,02 | 11,72 | 27,27 | 10,95 | 23,3 | 11 | 30 | 11,75 |  |  |
| CD14+ CD80/86+ | 18,85 | 45,56 | 30,33 | 35 | 23,6 | 77,56 | 11 | 30 | -26,71 | 0.03 | -49,95 - -3,4 |
| CD14+ TSLPR+ | 7,9 | 6,23 | 14,8 | 4,76 | 6,12 | 6,44 | 11 | 29 | 1,67 |  |  |
| CD14+ CD1a+ | 86,7 | 82,32 | 6,36 | 19,8 | 4,5 | 6,9 | 2 | 25 | 4,38 |  |  |
| CD14+ CD64+ | 0,665 | 1,17 | 0,5 | 1,39 | 0,35 | 0,65 | 2 | 23 | -0,51 |  |  |
| CD14+ CD163+ | 0,7 | 2,73 | 0,1 | 2,33 | 0,07 | 2,76 | 2 | 25 | -2,03 |  |  |
| CD3- CD56+ | 14,64 | 12,84 | 12,33 | 8,1 | 8,28 | 10,76 | 9 | 30 | 1,80 |  |  |
| CD3+ CD56+ | 8,22 | 13,16 | 7,26 | 10,49 | 10,62 | 11,67 | 9 | 30 | -4,94 |  |  |
| ILC2 | 1,13 | 1,39 | 0,86 | 0,89 | 0,25 | 0,88 | 9 | 30 | -0,26 |  |  |
| **Factors [ng / ml]** |  |  |  |  |  |  |  |  |  |  |  |
| TARC | 10.224 | 5.973 | 7324,3 | 5486,49 | 10771,67 | 7852,48 | 10 | 31 | 4250,95 |  |  |
| HGF | 3.856 | 1.805 | 2319,14 | 1959,63 | 3231,06 | 1275,04 | 9 | 30 | 2050,34 | 0.03 | 188,9 - 3911,7 |
| TGFß1 | 21895,4 | 46049,44 | 26881,83 | 29880,88 | 4840,78 | 45612,05 | 10 | 32 | -24154,04 | 0.03 | -45311 - -2997,0 |
| Periostin | 37400,04 | 67274,43 | 244446,18 | 39660,93 | 32104,36 | 34488,32 | 8 | 28 | -29874,39 | 0.02 | -5384,7 - -5907,06 |

**Table S1D Patients treated with Mesalazine / all other patients**

|  | **Mean** | | **SD** | | **IQR** | | **n** | | **Δ** | **p-value** | **95% CI** |
| --- | --- | --- | --- | --- | --- | --- | --- | --- | --- | --- | --- |
| **Leukocytes [FoP]** | **Mesalazine** | **other** | **Mesalazine** | **other** | **Mesalazine** | **other** | **Mesalazine** | **No Mesalazine** |  |  |  |
| CD19+ | 15,35 | 19,34 | 19,91 | 22,43 | 8 | 9 | 26 | 15 | -3,99 |  |  |
| CD19+ CD27+ IgD+ | 20,14 | 13,67 | 20,48 | 11,93 | 22,35 | 21,07 | 26 | 15 | 6,47 |  |  |
| CD19+ CD27+ IgD- | 76,1 | 77,66 | 22,08 | 24,63 | 26,55 | 26,4 | 26 | 15 | -1,56 |  |  |
| CD19+ 38+ | 22,79 | 26,89 | 26 | 34,74 | 43,23 | 48,97 | 26 | 15 | -4,10 |  |  |
| CD4+ | 21,29 | 27,93 | 13,03 | 11,15 | 11,7 | 14,6 | 26 | 15 | -6,64 |  |  |
| CD4+ CD25+ | 3,97 | 6,61 | 1,9 | 8,68 | 11,59 | 9,85 | 26 | 15 | -2,64 |  |  |
| CD8+ | 16,76 | 15,79 | 9,44 | 11,26 | 13,08 | 13,61 | 23 | 17 | 0,97 |  |  |
| CD11b+ | 9,62 | 12,55 | 7,85 | 7,33 | 5,96 | 8,24 | 26 | 15 | -2,93 |  |  |
| CD11b+ TSLPR+ | 18,48 | 18,4 | 23,25 | 25,54 | 16 | 15,71 | 26 | 15 | 0,08 |  |  |
| CD11b+ CD1a+ | 26,58 | 17,82 | 25,25 | 18,68 | 29,15 | 26,48 | 26 | 15 | 8,76 |  |  |
| CD11b+ CD80+ | 15 | 5,83 | 24 | 3,2 | 7,9 | 4,97 | 26 | 15 | 9,17 |  |  |
| CD11c+ | 0,67 | 1,14 | 0,46 | 0,85 | 0,6 | 1,1 | 26 | 15 | -0,47 | 0.05 | -1,0 - -0,0 |
| CD14+ | 5,62 | 6,45 | 7,3 | 4 | 6,59 | 4,04 | 26 | 15 | -0,83 |  |  |
| CD14+ CCR2+ | 75,76 | 76,81 | 23 | 29,14 | 24,87 | 21,05 | 26 | 15 | -1,05 |  |  |
| CD14+ CD80/86+ | 37,64 | 39,71 | 34,33 | 38,75 | 70,13 | 79,7 | 26 | 15 | -2,07 |  |  |
| CD14+ TSLPR+ | 8,15 | 6,36 | 11,58 | 5,36 | 6,58 | 7,9 | 26 | 15 | 1,79 |  |  |
| CD14+ CD1a+ | 85,25 | 78,85 | 12,06 | 25,62 | 5,25 | 6,1 | 16 | 17 | 6,40 |  |  |
| CD14+ CD64+ | 0,84 | 1,5 | 0,63 | 1,88 | 0,39 | 1,14 | 16 | 11 | -0,66 |  |  |
| CD14+ CD163+ | 2,63 | 2,63 | 2,42 | 2,25 | 3,43 | 1,31 | 16 | 11 | 0,00 |  |  |
| CD3- CD56+ | 12,59 | 15,18 | 8 | 11,47 | 7,7 | 18,2 | 25 | 14 | -2,59 |  |  |
| CD3+ CD56+ | 13,38 | 10,16 | 10,87 | 7,71 | 12,63 | 7 | 25 | 14 | 3,22 |  |  |
| ILC2 | 1,34 | 1,32 | 0,66 | 1,15 | 0,87 | 0,79 | 22 | 16 | 0,02 |  |  |
| **Factors [ng / ml]** |  |  |  |  |  |  |  |  |  |  |  |
| TARC | 6420,27 | 7762,17 | 6579,27 | 5698,28 | 8362,69 | 8039,47 | 23 | 18 | -1341,90 |  |  |
| HGF | 2230,49 | 2340,73 | 2050,61 | 2424,52 | 3270,27 | 2310,66 | 22 | 17 | -110,24 |  |  |
| TGFß1 | 38428,65 | 42791,59 | 27745,81 | 34916,53 | 43445,65 | 49557,92 | 24 | 18 | -4362,94 |  |  |
| Periostin | 60023,99 | 61492,03 | 47670,45 | 21939,75 | 42211,91 | 24219,64 | 21 | 15 | -1468,04 |  |  |

**Table S1E. Patients treated with immuno-suppressive drugs / all other patients.**

|  | **Mean** | | **SD** | | **IQR** | | **n** | | **Δ** | **p-value** | **95% CI** |
| --- | --- | --- | --- | --- | --- | --- | --- | --- | --- | --- | --- |
| **Leukocytes [FoP]** | **Immuno-suppressives** | **other** | **Immuno-suppressives** | **other** | **Immuno-suppressives** | **other** | **Immuno-suppressives** | **No Immuno-suppressives** |  |  |  |
| CD19+ | 38 | 12,15 | 29 | 14,74 | 51,35 | 6,99 | 7 | 34 | 25,85 | 0,05 | -1,0 - 52,78 |
| CD19+ CD27+ IgD+ | 6,17 | 22,44 | 9,99 | 22,58 | 3,41 | 23,65 | 7 | 34 | -16,27 | 0.006 | -27,45 - -5,08 |
| CD19+ CD27+ IgD- | 91,81 | 71,45 | 10,91 | 26,19 | 6,1 | 26,19 | 7 | 34 | 20,36 | 0.002 | 7,82 - 32,88 |
| CD19+ 38+ | 48,76 | 20,77 | 29,24 | 27,95 | 25,1 | 32,75 | 7 | 34 | 27,99 | 0.05 | 0,46 - 55,5 |
| CD4+ | 31,08 | 22,45 | 7,72 | 12,61 | 7,95 | 13,15 | 7 | 34 | 8,63 | 0.03 | 0,8 - 16,4 |
| CD4+ CD25+ | 9,43 | 3,9 | 13,75 | 2,02 | 3,3 | 1,18 | 7 | 34 | 5,53 |  |  |
| CD8+ | 25,92 | 14,32 | 8,28 | 9,37 | 7,85 | 10,96 | 7 | 34 | 11,60 | 0.008 | 4,0 -19,78 |
| CD11b+ | 10,31 | 11,17 | 6,56 | 7,81 | 9,75 | 7,64 | 7 | 34 | -0,86 |  |  |
| CD11b+ TSLPR+ | 37,7 | 14,15 | 32,37 | 19,98 | 46,85 | 12,2 | 7 | 34 | 23,55 |  |  |
| CD11b+ CD1a+ | 17,57 | 24,16 | 32,3 | 21,15 | 12,81 | 21,95 | 7 | 34 | -6,59 |  |  |
| CD11b+ CD80+ | 14,85 | 10,9 | 32,23 | 16,54 | 3,27 | 4,99 | 7 | 34 | 3,95 |  |  |
| CD11c+ | 1,32 | 0,77 | 0,85 | 0,61 | 1,02 | 0,68 | 7 | 34 | 0,55 |  |  |
| CD14+ | 5,57 | 6,24 | 4,8 | 6,64 | 6,08 | 7,69 | 7 | 34 | -0,67 |  |  |
| CD14+ CCR2+ | 77,75 | 76,16 | 18,9 | 26,08 | 27 | 19,85 | 7 | 34 | 1,59 |  |  |
| CD14+ CD80/86+ | 20,52 | 41,49 | 24,19 | 36,27 | 41,56 | 77,67 | 7 | 34 | -20,97 |  |  |
| CD14+ TSLPR+ | 9,84 | 6,84 | 18,13 | 7,29 | 6,8 | 6,78 | 7 | 34 | 3,00 |  |  |
| CD14+ CD1a+ | 83,4 | 79,64 |  | 24,55 |  | 7,4 | 1 | 26 | 3,76 |  |  |
| CD14+ CD64+ | 1,61 | 1,11 |  | 1,36 |  | 0,49 | 1 | 26 | 0,50 |  |  |
| CD14+ CD163+ | 2 | 2,99 |  | 2,9 | 0 | 3,47 | 1 | 26 | -0,99 |  |  |
| CD3- CD56+ | 17,65 | 13,26 | 13,71 | 8,48 | 20,46 | 10,9 | 6 | 33 | 4,39 |  |  |
| CD3+ CD56+ | 8,36 | 12,72 | 7,11 | 11,16 | 10,49 | 14,22 | 6 | 33 | -4,36 |  |  |
| ILC2 | 0,91 | 1,41 | 0,48 | 0,92 | 0,52 | 0,93 | 6 | 32 | -0,50 | 0.07 |  |
| Factors [ng / ml] |  |  |  |  |  |  |  |  |  |  |  |
| TARC | 12055,61 | 6144,33 | 3077,32 | 6174,33 | 2684,63 | 6640,84 | 6 | 35 | 5911,28 | 3E-03 | 2392,1 - 9430,4 |
| HGF | 3697,63 | 2069,85 | 2225,27 | 2139,94 | 2969,18 | 2340,91 | 5 | 34 | 1627,78 |  |  |
| TGFß1 | 17610,67 | 44079,78 | 25195,68 | 30166,39 | 4610,11 | 49344,76 | 6 | 36 | -26469,11 | 0.05 | -53106,6 - 168,3 |
| Periostin | 45944,37 | 63005,24 | 15079,72 | 40842,95 | 10212,66 | 37427,42 | 5 | 31 | -17060,87 |  |  |

**Table S2 Analysis of human Colon**

| **Diagnosis** | **Leucocyte** | **mean** | **sd** | **IQR** | **n** | **Δ** | **p-value** | **95% CI** |
| --- | --- | --- | --- | --- | --- | --- | --- | --- |
| Non UC | CD4+ | 2,93 | 1,51 | 1 | 5 | 4,46 |  |  |
| UC | CD4+ | 7,39 | 5,21 | 7,3 | 4 |  |  |  |
| Non UC | CD8+ | 4,19 | 2,8 | 2,8 | 5 | 0,85 |  |  |
| UC | CD8+ | 5,04 | 6,2 | 4,5 | 4 |  |  |  |
| Non UC | CD11b+ | 1,53 | 0,85 | 0,95 | 5 | 4,87 |  |  |
| UC | CD11b+ | 6,4 | 7 | 7,31 | 4 |  |  |  |
| Non UC | CD11c+ | 0,28 | 0,15 | 0,22 | 5 | -0,25 |  |  |
| UC | CD11c+ | 0,03 | 0,04 | 0,05 | 4 |  |  |  |
| Non UC | CD14+ | 1,9 | 1,37 | 2 | 5 | 0,7 |  |  |
| UC | CD14+ | 2,6 | 0,48 | 0,79 | 4 |  |  |  |
| Non UC | CD3+ CD56+ | 31,63 | 23,04 | 25,5 | 5 | 40,74 | 0,01 | -68,9 - -12,5 |
| UC | CD3+ CD56+ | 72,37 | 6 | 3,8 | 4 |  |  |  |
| Non UC | CD3- CD56+ | 36,68 | 19,13 | 16,1 | 4 | 22,72 |  |  |
| UC | CD3- CD56+ | 59,4 | 28,54 | 17,1 | 5 |  |  |  |
| Non UC | CD11b+ CD1a+ | 22,54 | 11,65 | 14,8 | 5 | 59,36 | 0,0002 | -78,3 - -40,5 |
| UC | CD11b+ CD1a+ | 81,9 | 11,78 | 8,87 | 4 |  |  |  |
| Non UC | CD11b+ TSLPR+ | 3,56 | 2,86 | 2,3 | 5 | 7,44 |  |  |
| UC | CD11b+ TSLPR+ | 11 | 11 | 7,35 | 4 |  |  |  |
| Non UC | CD14+ CD1a+ | 34,8 | 11,8 | 10,3 | 5 | 39,2 | 0,08 |  |
| UC | CD14+ CD1a+ | 74 | 32 | 26,9 | 4 |  |  |  |
| Non UC | CD14+ CD64+ | 34,2 | 38,7 | 58,46 | 5 | 16,6 |  |  |
| UC | CD14+ CD64+ | 50,8 | 25,27 | 36,3 | 4 |  |  |  |
| Non UC | CD14+ TSLPR+ | 7,17 | 9,11 | 8,32 | 5 | 5,59 |  |  |
| UC | CD14+ TSLPR+ | 12,76 | 18,82 | 17,78 | 4 |  |  |  |
| Non UC | CD14+ CD163+ CD206+ | 42,72 | 49,71 | 87,87 | 5 | -15,26 |  |  |
| UC | CD14+ CD163+ CD206+ | 27,46 | 20,84 | 15,93 | 4 |  |  |  |

**Table S4. Antibodies used in flow cytometric analysis**

| Surfacemarker (anti human) | Colour | Clone |
| --- | --- | --- |
| CD19 | Peridine-chlorophyll-protein complex cyanine dye (PerCP-Cy^TM^ 5.5) | HIB19 |
| CD38 | Phycoerythrin (PE) | HB-7 |
| CD27 | Phycoerythrin cyanin (Pe-Cy7) | LG.3A10 |
| IgD | Fluorescin isothiocyanate (FITC) | LA6-2 |
| CD4 | Allophycocyanin (APC)- Cy7 | OKT4 |
| CD44 | PE | BJ18 |
| CD62L | FITC | DREG-56 |
| CD8 | PerCP-Cy^TM^ 5.5 | HIT8a |
| CD103 | APC | Ber-ACT8 |
| CD25 | PE-Cy7 | BC96 |
| CD294 (CRTH2) | APC | BM16 |
| CD14 | APC-Cy7 | HCD14 |
| CCR2 | PE-Cy7 | K036C2 |
| CD80/86 | PerCP-Cy^TM^ 5.5 | IT2.2 |
| TSLPR | APC | 1B4 |
| CD1a (biotin)/secundary Ab streptavidin | FITC | HI149 |
| CD64 | PerCP-Cy^TM^ 5.5 | 10.1 |
| CD163 | FITC | GHI/61 |
| CD16 | PE | 3G8 |
| CD11b | APC-Cy7 | M1/70 |
| CD11c | PE-Cy7 | 3.9 |
| CD3 | APC-Cy7 | HIT3a |
| CD56 | FITC | HCD56 |
| CD94 | PE | DX22 |
| KIR | PE-Cy7 | DX27 |
| CD127 | PerCP-Cy^TM^ 5.5 | A019D5 |
